# T-cell intracellular antigens function as tumor suppressor genes

**DOI:** 10.1038/cddis.2015.43

**Published:** 2015-03-05

**Authors:** C Sánchez-Jiménez, M D Ludeña, J M Izquierdo

**Affiliations:** 1 Centro de Biología Molecular Severo Ochoa, Consejo Superior de Investigaciones Científicas, Universidad Autónoma de Madrid (CSIC/UAM), C/ Nicolás Cabrera 1, Madrid, Spain; 2 Facultad de Medicina, Departamento de Biología Celular y Patología, Universidad de Salamanca-Hospital Universitario de Salamanca, C/ Paseo de San Vicente 58–182, Salamanca, Spain

## Abstract

Knockdown of T-cell intracellular antigens TIA1 and TIAR in transformed cells triggers cell proliferation and tumor growth. Using a tetracycline-inducible system, we report here that an increased expression of TIA1 or TIAR in 293 cells results in reduced rates of cell proliferation. Ectopic expression of these proteins abolish endogenous TIA1 and TIAR levels via the regulation of splicing of their pre-mRNAs, and partially represses global translation in a phospho-eukaryotic initiation factor 2 alpha-dependent manner. This is accompanied by cell cycle arrest at G1/S and cell death through caspase-dependent apoptosis and autophagy. Genome-wide profiling illustrates a selective upregulation of p53 signaling pathway-related genes. Nude mice injected with doxycycline-inducible cells expressing TIA1 or TIAR retard, or even inhibit, growth of xenotumors. Remarkably, low expressions of TIA1 and TIAR correlate with poor prognosis in patients with lung squamous cell carcinoma. These findings strongly support the concept that TIA proteins act as tumor suppressor genes.

T-cell intracellular antigens (TIA) are multifunctional proteins that operate as ancient DNA/RNA *trans*-acting regulators to broaden transcriptome and proteome diversity.^1, 2, 3^ TIA proteins modulate many regulatory aspects of RNA metabolism. In the nucleus, they control transcription by interacting with DNA and RNA polymerase II.^4, 5, 6^ Further, they facilitate the splicing of pre-mRNAs by improving the selection of constitutive and atypical 5′ splice sites.^7, 8, 9, 10, 11^ In the cytoplasm, they regulate localization, stability and/or translation of eukaryotic mRNAs by binding to the 5′ and/or 3′-untranslated regions.^11, 12, 13, 14, 15, 16, 17, 18, 19, 20^ Consequently, these regulators impact prevalently on the cellular biology of specific RNAs and proteins, defining their fate into ribonucleoprotein complexes.^1, 2, 7, 8, 9, 10, 11, 12, 13, 14, 15, 16, 17, 18, 19, 20^

TIA proteins are known to target genes involved in essential biological functions, including embryogenesis, death/survival, proliferation/differentiation, inflammation, environmental stress, viral infection and tumorigenesis.^1, 2, 12, 13, 14, 15, 16, 17, 18, 19, 20, 21, 22, 23, 24, 25^

Though the known importance of TIA proteins in inflammation and the stress response, their role(s) in proliferation/differentiation and survival/death responses is less understood. We previously reported that a reduction of TIA expression in HeLa cells enhances proliferation, invasion and tumor growth.^21, 26^ Further, TIA proteins are downregulated in a subset of human epithelial tumors. These observations suggest that TIA proteins could act as inhibitors of tumorigenesis.^21, 26^ However, these capabilities have never been established. Thus, we developed an inducible expression model to evaluate this possibility and explore the mechanisms associated to these novel traits. Herein, we clearly showed that TIA proteins function as cell growth and tumor suppressor genes. In addition, TIA proteins may have potential as biomarkers and prognosis factors in human lung cancer.

## Results

### Ectopic expression of TIA proteins specifically down-regulates endogenous TIA proteins

**Figure 1:**
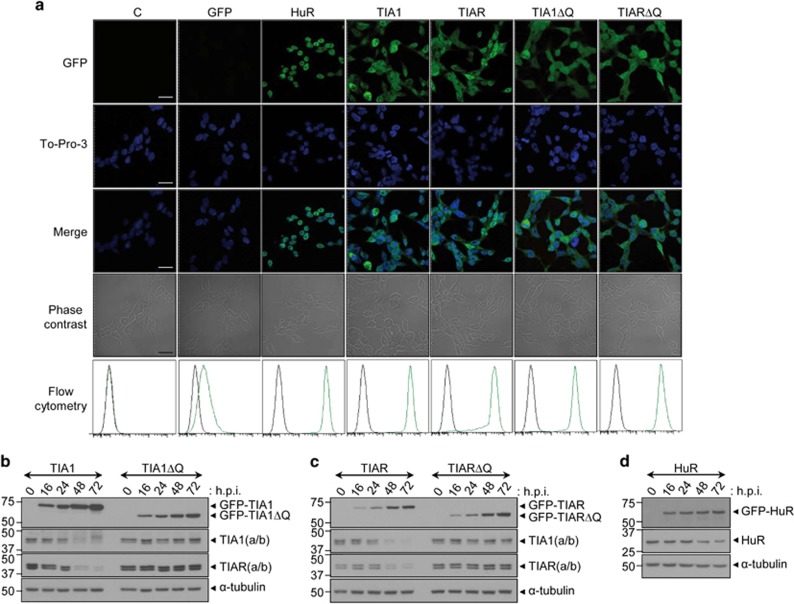
Expression patterns of GFP-tagged proteins in tetracycline-induced FT293 cells. (**a**) Fluorescence images from FT293 cells expressing indicated proteins by confocal microcopy. The middle panels are the sum of GFP and To-Pro-3 images (Merge). Phase contrast photographs are also shown. Scale bars represent 20 *μ*m. The fluorescence levels were analyzed by flow cytometry (**b**–**d**) Time-course and expression profiles of ectopic and endogenous TIA1, TIAR and HuR proteins in indicated FT293 cells by immunoblotting

The Flp-In T-REx System was used to generate inducible FT293 cell lines expressing GFP, TIA1, TIAR or HuR proteins, as well as truncated versions of TIA1 and TIAR lacking the C-terminal Q-rich (ΔQ) domains. The subcellular localization and protein expression patterns from different GFP-tagged constructs, expressed in FT293 cells for 3 days, were examined by confocal microscopy and western blotting. Results showed that the expression of tetracycline-inducible TIA1, TIA1ΔQ, TIAR, TIARΔQ and HuR proteins was consistent with their well-established patterns of nucleocytoplasmic and nuclear (with nucleolar exclusion) localizations for endogenous TIA1, TIAR and HuR, respectively (Figure 1a and refs 27, 28). Compared with the oxidative stress agent arsenite, analysis demonstrated that cells overexpressing TIA proteins did not trigger the massive formation of stress granules (Figure 1a and Supplementary Figure S1). However, ectopic TIA1 or TIAR expression promoted the depletion of endogenous TIA proteins (Figures 1b and Supplementary Figure S2A). This depletion occurred in a ΔQ domain-dependent manner (Figures 1b and Supplementary Figures S2B and D) and was associated solely with expression of TIA proteins as the ectopic expression of TIA1 or TIAR did not alter endogenous HuR expression (Figures 1b and d and Supplementary Figure S2). Similarly, the endogenous expression of TIA1 and TIAR was unaffected by ectopic HuR expression, whereas the endogenous HuR levels were decreased (Supplementary Figure S2A and ref. 29). Importantly, *α*-tubulin levels were not significantly changed (Figures 1b and d and Supplementary Figure S2A). These results are consistent with the notion of regulatory crosstalk between TIA proteins.

Previous reports showed that TIA1 and TIAR activate splicing of alternative exons with weak 5' splice sites followed by a U-rich stretch on their own pre-mRNAs.^10, 30^ We sought to verify this observation in our gain-of-function model. We found that the expression of TIA1 or TIAR, but not their corresponding ΔQ versions, activated splicing of some unusually used alternative exons and also cryptic 5′ splice sites, in place of the original splice sites, on TIA1 (Supplementary Figures S2B, D and S3) and TIAR (Supplementary Figures S2C, D and S3) pre-mRNAs. These novel RNA isoforms contained exons with premature stop codons that, when included, interrupted their open reading frames, resulting in decreased levels of these factors and facilitating their elimination by nonsense-mediated mRNA decay.^10, 30^ Thus, ectopically expressed TIA proteins function as splicing regulators to reprogram the expression of endogenous TIA proteins, validating their putative contributions to cellular pathways associated with cell and tumor growth.

### Expression of TIA proteins suppresses cell proliferation

**Figure 2:**
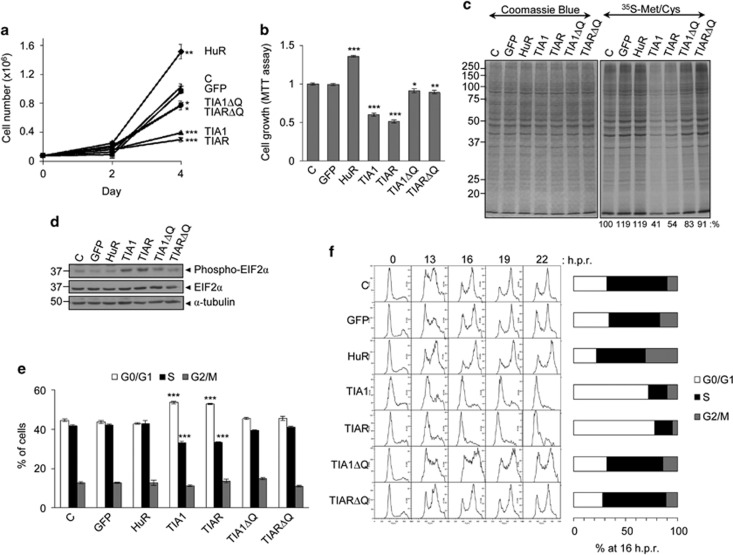
Expression of TIA proteins suppresses cell proliferation leading to cell cycle arrest at the G1/S phase. (**a**) FT293 cells expressing indicated proteins were counted on the days indicated for 4 days (**b**) Cells grown for 4 days were monitored by methyl thiazolyl tetrazolium (MTT) assay. The represented values were normalized and expressed relative to control (c), whose value was fixed arbitrarily to 1 (**c**) Nascent translation rates of total proteins were determined by incubation of above FT293 cells in the presence of ^35^S-methionine/-cysteine (Met/Cys) mix. The relative translational rates were estimated as ratio ^35^S-Met/Cys label versus Coomassie Blue staining. The values are indicated as percentages referred to control (**c**) (**d**) Analysis of the phosphorylation status of Ser 51 on eukaryotic initiation factor 2 alpha subunit (eIF2*α*) (**e**) Analysis of cell cycle phases by flow cytometry (**f**) FT293 cells expressing TIA proteins showed cell cycle arrest at the G1/S phase. The indicated FT293 cells were synchronized at G1/S by hydroxyurea blockage for 30 h, released and quantified at 16 h post release. In all cases, the represented values are means±S.E.M. (*n*=3–6; *P<0.05; **P<0.01; ***P<0.001)

We and others have demonstrated that decreased expression of TIA proteins increases proliferation of transformed cells.^17, 18, 20, 21, 22^ Overexpression of TIA1 or TIAR correlated with a significant reduction in cell proliferation, as measured by total cell counting and methyl thiazolyl tetrazolium activity. Conversely, the overexpression of HuR resulted in increased cell proliferation (Figures 2a and b), consistent with their well-documented regulatory roles in cell and tumor growth.^31, 32^ Ectopic expression of ΔQ-truncated TIA proteins resulted in a reduced inhibitory effect compared with full-length proteins (Figures 2a and b). Estimation of *de novo* protein synthesis by [^35^S]-methionine and -cysteine incorporation corroborated these findings (Figure 2c), and indicated relevant roles for each TIA protein as translational repressors. Indeed, these results showed a significant inhibition of global translational rates (~40–50%), which correlated with phosphorylation of eukaryotic initiation factor 2 alpha (eIF2*α*; Figure 2d). Therefore, sustained expression of TIA1 or TIAR reduces cell proliferation, in part, because the global rate of cellular translation is inhibited.

To determine the impact of TIA expression on the cell cycle, the percentage of cells in each cell cycle phase was determined by flow cytometry. Expression of full-length TIA1 or TIAR but not GFP, HuR, TIA1ΔQ or TIARΔQ, resulted in a significant increase of cells in G0/G1, with a reciprocal decrease in the S phase (Figure 2e). In addition, we analyzed the cell cycle profile of cells released after arrest at the G1/S phase with hydroxyurea. Compared with control cells, TIA1- or TIAR-expressing cells exhibited a major cell cycle block at G1/S (Figure 2f), indicative of specific cell cycle arrest (∼80% at 16 h post release) in the transition from G1 to S. Finally, we tested whether TIA1 or TIAR expression provoked cell senescence by assessing the expression of the senescence-associated *β*-galactosidase. However, there was no evidence of positive staining in FT293 cells expressing TIA1 or TIAR (Supplementary Figure S4). These data suggest that the expression of TIA leads to the accumulation of cells in G1/S and reduced proliferation.

### Expression of TIA proteins increases cell death

**Figure 3:**
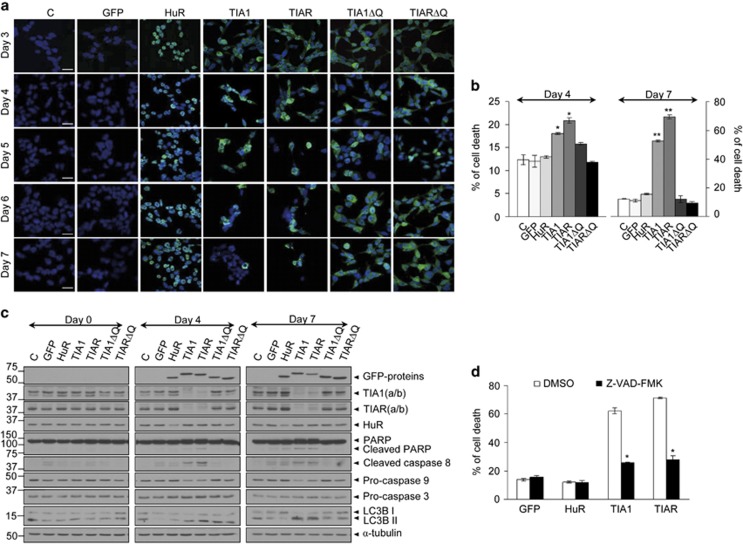
Expression of TIA proteins leads to slow cell death mediated by caspase-dependent apoptosis and late autophagy. (**a**) Analysis of cellular morphology and viability of FT293 cells for 3–7 days. Scale bars represent 20 *μ*m (**b**) Quantification of cell death rates at 4 and 7 days. The represented values are means±S.E.M. (*n*=3–5; *P<0.01; **P<0.001) (**c**) Western blot analysis of cell death markers at 4 and 7 days (**d**) Cell death by apoptosis occurs in a caspase-dependent way. FT293 cells were grown for 4 days and then treated for 4 days further with DMSO or caspase inhibitor Z-VAD-FMK. The represented values are means±S.E.M. (*n*=2; *P<0.01)

Given these growth-restricted phenotypes, cell lines were analyzed by microscopy for an extended period (3–7 days post induction). Sustained TIA1 and TIAR expression for 4 days was associated with obvious deleterious changes in cellular and nuclear morphology that became dramatic after 7 days in culture (Figure 3a and Supplementary Figure S4), indicating the existence of additional mechanisms that limit cell growth. We therefore measured cell death by flow cytometry. We found a significant increase in the percentage of cells undergoing cell death at 4 days, which was further elevated at 7 days (Figure 3b). Analysis of molecular markers related to apoptotic and autophagic phenotypes, as cleaved poly (ADP-ribose) polymerase and activated caspase-3, 8 and 9, or processed LC3B-II, respectively, confirmed the evolution of cell death at 4 and 7 days (Figure 3c), and were consistent with the existence of cell death events associated with slow apoptosis and late autophagy (Figure 3c). In addition, treatment of FT293 cells expressing GFP, HuR, TIA1 or TIAR with the broad-spectrum caspase inhibitor Z-VAD-FMK for 4 days resulted in a significant prevention of apoptosis in TIA1- and TIAR-expressing cells (Figure 3d). Collectively, these findings strongly suggest that sustained TIA1 or TIAR expression inhibits cell proliferation, favors cell cycle arrest at G1/S and triggers cell death through slow caspase-dependent apoptosis and late autophagy.

### Genome-wide profiling analysis of FT293 cells

**Figure 4:**
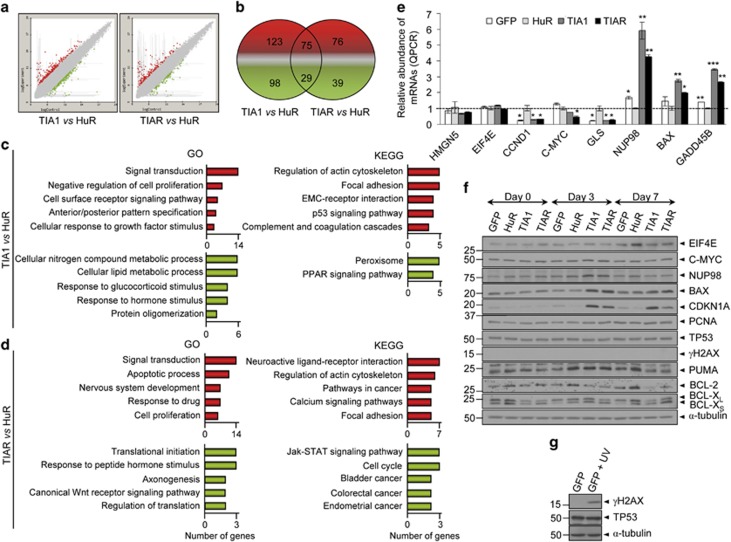
Characterization of the transcriptomes associated to FT293 cells expressing TIA1, TIAR or HuR proteins. (**a**) MA plot representation of the distribution of up- (spots in red) and downregulated (spots in green) RNAs (−2 ⩾ fold-change ⩽ 2; FDR<0.05) in TIA1- or TIAR- versus HuR-expressing FT293 cells, respectively (**b**) Venn diagrams depicting the numbers of genes that were upregulated (red) or downregulated (green) as well as shared between both categories (**c** and **d**) Top-five categories of biological processes and pathways in TIA1- or TIAR- versus HuR-expressing FT293 cells, respectively. Histograms represent the numbers of up- (red) and downregulated (green) genes using the GO and the KEGG pathway databases (*P*<0.05), respectively (**e**) Quantification of relative expression levels of indicated mRNAs by qPCR. The represented values were normalized and expressed relative to GAPDH. The represented values are means±S.E.M. (*n*=2; *P<0.05; **P<0.01; ***P<0.001) (**f** and **g**) Western blot analysis from indicated FT293 cells at 0, 3 and 7 post-induction days (**f**) and FT293 cells either control or irradiated with ultraviolet light (**g**)

To gain molecular insights into the emergence of these cellular phenotypes, we surveyed the transcriptome of FT293 cells 3 days after treatment with tetracycline, which was the earliest time point to observe phenotypic differences between HuR- and TIA1-expressing cells before the onset of cellular death. Global RNA expression profiles of TIA1, TIAR and HuR cells and control (GFP) cells were obtained using a human genome-wide microarray. As shown in Figure 4a, TIA1 or TIAR gain-of-function cells displayed moderate differences in gene expression profiles when compared with the RNA profile of HuR-expressing cells. A total of 325 (of which 198 and 127 were up- and downregulated, respectively) and 219 (of which 151 and 68 were up- and downregulated, respectively) RNAs were differentially expressed (−2 ⩾ fold-change ⩾ 2; FDR limma <0.05) in TIA1- or TIAR- versus HuR-expressing cells, respectively (Figures 4a and Supplementary Figures S4 and S5). Of those, 75 up- and 29 downregulated RNAs were common to both TIA1- and TIAR-expressing cells (Figure 4b and Supplementary Figure S5). These results indicate that TIA proteins regulate/modulate both specific and overlapping aspects of the human transcriptome, according to previous findings.^11, 18, 22, 25^

To understand the functional relevance of differentially expressed genes (DEGs), gene ontology (GO) and Kyoto Encyclopedia of Genes and Genomes (KEGG) classification analysis was performed. GO analysis identified the main biological processes of DEGs modulated by TIA proteins (*P*<0.05; Figures 4c and d and Supplementary Figure S6). GO categories related to signal transduction, negative regulation of cell proliferation, cell surface receptor signaling pathway, anteroposterior pattern specification and cellular response to growth factor stimulus were among the top five enriched categories in upregulated genes by TIA1 expression (Figure 4c and Supplementary Figure S6). Further, GO categories associated with cellular nitrogen compound and lipid metabolism processes, responses to glucocorticoid and hormone stimuli and protein oligomerization were especially prevalent among downregulated genes.

KEGG database analysis identified enrichment of specific pathways in functionally regulated gene groups (Figure 4c and Supplementary Figure S6). Several KEGG pathways were significantly enriched (*P*<0.05) in upregulated genes, including those involved in the regulation of actin cytoskeleton, focal adhesion, EMC–receptor interaction, p53 signaling and complement and coagulation cascades. KEGG pathways significantly enriched (*P*<0.05) in downregulated genes included peroxisome and PPAR signaling. Collectively, these results demonstrate major alterations in programs controlling cell proliferation and signal transduction, together with pathways regulating specific cellular stress, cell death, cell cycle and metabolic responses that may modulate the cellular phenotypes associated with TIA1 overexpression.

A similar analysis was performed in TIAR-expressing cells. The top five GO categories (*P*<0.05) associated with upregulated genes included those related to signal transduction, apoptosis, nervous system development and response to drug and cell proliferation (Figure 4d and Supplementary Figure S6). GO categories (*P*<0.05) of downregulated genes highlighted genes involved in translational initiation, response to peptide hormone stimuli, axonogenesis, canonical Wnt receptor signaling and regulation of translation. KEGG data (*P*<0.05) for upregulated genes in TIAR-expressing FT293 cells were associated with genes related to neuroactive-ligand receptor interaction, regulation of actin cytoskeleton, cancer pathways, calcium signaling and focal adhesion (Figure 4d and Supplementary Figure S6). KEGG categories for downregulated genes in TIAR-expressing cells were related to Jak–STAT signaling, cell cycle and bladder, colorectal and endometrial cancers. To question whether increased expression of TIA proteins was associated with the regulation of common gene clusters, we used GO and KEGG database analysis to examine all genes that were identified as up- or downregulated by both TIA1 and TIAR overexpression (Supplementary Figure S6). Results indicated that shared TIA1 and TIAR genes were related to regulation of cell proliferation, apoptosis and p53 signaling. Thus, TIA proteins modulate specific and overlapping negative aspects of cell proliferation, apoptosis, the cell cycle and cancer-related pathways, which may contribute to the cellular phenotypes associated with sustained expression of TIA proteins.

### Validation of microarray-predicted changes in gene expression

To validate the microarray-generated data, we measured the expression of relevant genes by qPCR. As expected, we detected the down- and upregulation of mRNAs in FT293 cells expressing GFP, HuR, TIA1 or TIAR proteins, respectively (Figure 4e). Some representative genes were additionally quantified by western blotting. Protein expression of EIF4E, C-MYC, nucleoporin 98 kDa (NUP98) and BCL-2-associated X protein (BAX) was consistent with the results from PCR (Figure 4f). Interestingly, several of these proteins are paradigmatic targets of the p53 tumor suppressor pathway.^33, 34, 35, 36, 37, 38^ Indeed, the G1/S cell cycle regulator cyclin-dependent kinase inhibitor 1A (CDKN1A), a major p53-regulated inhibitor of cyclin-dependent kinases,^33, 34, 35, 36, 37, 38^ was significantly induced by TIA expression. Similarly, the apoptotic inducers BAX, PUMA and BCL-X_S_ together with caspase 8 and 9 were activated (Figure 3c), whereas the survival factors BCL-2 and BCL-X_L_ were downregulated (Figure 4f). Unexpectedly, the protein expression levels of p53 and phosphorylated *γ*H2AX (at Ser139) protein, a specific marker of damaged DNA,^39, 40, 41^ were similar in cells expressing GFP, HuR, TIA1 or TIAR (Figures 4f and g). Collectively, these observations suggest the induction of a subset of gene targets associated with p53 signaling pathway, which may not be directly related to the DNA damage response but may contribute to the establishment of previous cellular phenotypes.

### TIA proteins modulate different regulatory layers of gene expression

**Figure 5:**
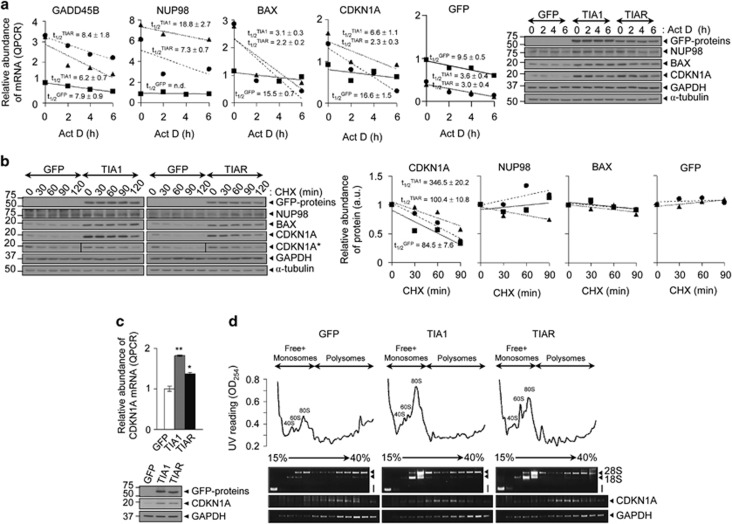
Expression of TIA proteins alters transcription, mRNA turnover, translation and protein stability. (**a**) DNA transcription was inhibited by the addition of Act D (5 *μ*g/ml) to the indicated FT293 cells. Steady-state mRNA levels were quantified by qPCR and represented at indicated times. The represented values corresponding to half-lives (*t*_1/2_ in the hour range) of mRNAs calculated from semilogaritmic plots to the first-order rate kinetics of the decay of mRNA. Validation of relative expression levels for selected proteins by western blotting (**b**) Translation elongation was inhibited by the addition of CHX (50 *μ*g/ml) to the indicated FT293 cells. Steady-state protein levels were analyzed by immunoblot and represented at indicated times. The resulting value corresponding to half-life (*t*_1/2_ in the min range) of CDKN1A protein was calculated as before (**c**) The relative expression levels of CDKN1A mRNA and protein in indicated FT293 cells were determined by qPCR and immunoblotting (**d**) Polysome profiling using sucrose gradients from indicated FT293 cells. The quality of polysomal preparations was verified by quantification of optical density at 254 nm and electrophoresis analysis in agarose gels of RNA content in each fraction. The fractions from top to bottom with increased density (15–40% sucrose) are identified as free-monosomes (fractions 1–5; identified as RNP, 40S, 60S and 80S), light polysomes (fractions 6–9) and heavy polysomes (fractions 10–12). Ribosomal 18S and 28S bands and initial input (I) are also shown. Distribution of CDKN1A and GAPDH mRNAs across sucrose gradients was verified by semiquantitative RT-PCR analysis

To better define the mechanisms underlying the transcriptomic patterns induced by TIA proteins, FT293 cells expressing GFP, TIA1 or TIAR were treated with actinomycin D (Act D). Results from steady-state mRNA decay analysis of growth arrest and DNA-damage-inducible, beta (GADD45B), NUP98, BAX, CDKN1A and GFP-tagged mRNAs suggested a predominant effect on mRNA transcription rather than mRNA stability (Figure 5a), whereas NUP98 mRNA decay suggested that stability could be exerting a specific regulatory role. Some of these proteins were also quantified by western blotting. In contrast to that observed in mRNA decay analysis, results showed that steady-state protein levels of NUP98, BAX, CDKN1A and GFP-tagged proteins were relatively constant after Act D treatment (Figure 5a), suggesting the existence of additional regulatory layers. Using the TIA-*in vivo* UV-crosslinking and immunoprecipitation (TIA-iCLIP) database,^11^ we assessed whether the upregulated p53-related targets had experimental TIA-binding sites. Interestingly, the 3′-untranslated regions of some of these mRNAs contain several sites and motifs for TIA binding (Supplementary Figure S7A); indeed, the TIA-associated NUP98 iCLIP profile was notable as its pre-mRNA sequence displayed multiple interaction sites with these proteins. Thus, we tested whether ectopically expressed TIA proteins could bind some of these mRNAs. Inducible FT293 cell extracts expressing GFP, GFP-TIA1, GFP-TIAR or GFP-HuR were immunoprecipitated with an anti-GFP monoclonal antibody coupled to magnetic beads and the immunoprecipitated mRNAs were analyzed by qPCR. The best candidates recovered from TIA1 and TIAR immunoprecipitates were NUP98»GADD45B=BAX=CDKN1A mRNAs (Supplementary Figure S7B), suggesting that TIA proteins may modulate the posttranscriptional status of these mRNAs (in particular, NUP98).

Given that the results for mRNA synthesis and/or stability did not conclusively explain the steady-state protein levels, as FT293 cells expressing TIA1 or TIAR present partial inhibition of global cell translation, we estimated the contribution of *de novo* protein synthesis and/or protein stability in cycloheximide (CHX)-treated FT293 cells (Figure 5b). Results showed a target-dependent differential effect of the inhibitor on *de novo* protein synthesis (Figure 5b). Whereas steady-state levels of NUP98 and BAX were refractory to CHX, demonstrating their intrinsic stability, the effects on CDKN1A expression, despite an increased half-life in TIA1 and TIAR-expressing FT293, were more evident, indicating that protein stability is an important factor (Figure 5b). As CDKN1A mRNA expression was relatively modest at the state-steady mRNA levels (Figure 5c), and showed a reduced protein half-life (Figure 5b), whereas it was highly found in TIA1 and TIAR-expressing FT293 cells (Figures 4 and 5), we tested the contribution of translational rates of this mRNA. Cytoplasmic extracts were fractionated through sucrose gradients, with the lightest components appearing at the top (fractions 1 and 2), small (40S) and large (60S) ribosomal subunits, and monosomes (80S) in fractions 3–6, and progressively larger polysomes in fractions 7–12 (Figure 5d). Compared with control GFP cells, results showed a partial translational repression in TIA1 and TIAR-expressing cells illustrated by the accumulation of 80S ribosomes (Figure 5d), in agreement with previous results (Figures 2c and d). The distribution of CDKN1A mRNA relative to the housekeeping gene glyceraldehyde-3-phosphate dehydrogenase (GAPDH) was measured by semiquantitative RT-PCR analysis in all fractions and total RNA (I). We found an enrichment of GAPDH mRNA in heavy polysomes versus free+monosomes fractions in the three FT293 cell lines analyzed. In contrast, CDKN1A mRNA was sedimented on lighter polysomes in cells expressing TIA1 or TIAR. This result suggests that ectopic expression of TIA proteins alters the global translational machinery and efficiency of specific mRNAs (Figure 5d), indicating that CDKN1A expression is regulated predominantly at the transcriptional and posttranslational levels.

To determine whether this process was reversible, FT293 cells growing in the presence of tetracycline and expressing TIA1 or TIAR for 4 days were switched to tetracycline-free medium for a further 4 days. We detected retrieval of several molecular markers at the basal steady-state expression levels (Supplementary Figure S8A). Further, FACS analysis showed that the transition from G1 cell cycle arrest to S and G2/M was reactivated (Supplementary Figure S8A). However, this outcome was not reproduced by silencing CDKN1A using RNA interference (Supplementary Figures S8B and C). Collectively, these observations suggest that the gene expression patterns and cell phenotypes detected in FT293 cells expressing TIA1 or TIAR could result from reversible and overlapping controls, implicating several molecular events at the transcriptional and posttranscriptional regulatory layers.

### TIA proteins can function as tumor suppressor genes

**Figure 6:**
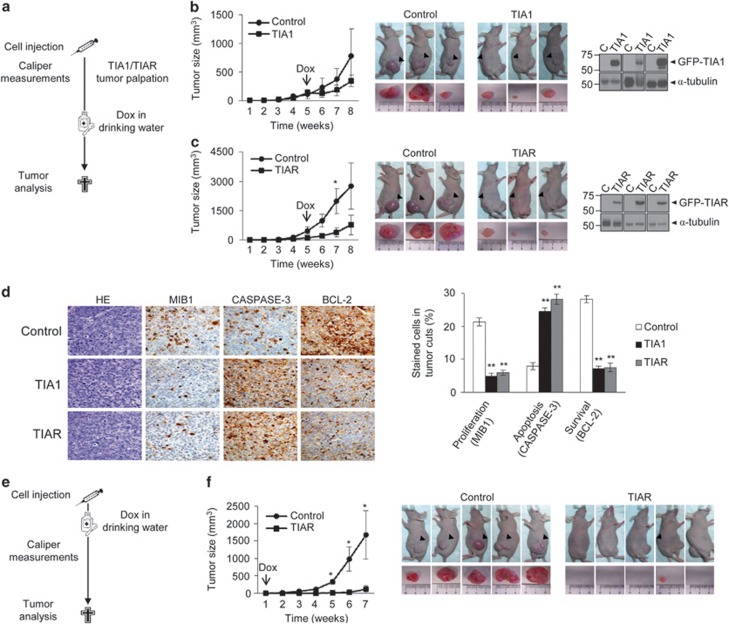
Expression of TIA proteins blocks *in vivo* tumor growth. (**a**–**c**) Workflow (**a**): control FT293 cells and TIA1- (**b**) or TIAR- (**c**) expressing FT293 cells (5 × 10^6^) were injected s.c. into the hind legs of female nude mice. Five to six mice were used for each cell pair. These cells were allowed to form tumors for 3–7 weeks before introduction of Dox into drinking water. Animals were killed 8 weeks after initial tumor palpation. Progression of tumor size after inoculation and Dox treatment was plotted. Tumor size is shown as mean±S.E.M. (*n*=5–6; *P<0.05). Representative photographs are shown for mice cohorts and tumors arising in the same mice. Expression of GFP-tagged proteins into xenograft tumors was verified by immunoblotting (**d**) Histological sections and immunohistochemical characterization of above xenograft tumors. Sections were stained with HE and other were immunostained with anti-MIB1/Ki67 (as proliferation index), anti-caspase-3 (as apoptosis indicator) and anti-BCL-2 (as survival indicator) antibodies. Scale bar represents 25 *μ*m. The number of stained cells as percentage in tumor cuts was quantified in at least 10 different cellular fields and represented values are the mean±S.E.M. (*n*=10; **P<0.001) (**e** and **f**) Workflow (**e**): control FT293 cells and TIAR- expressing FT293 cells (5 × 10^6^) were injected as before. These cells were allowed to form tumors for 1 week before introduction of Dox into drinking water. Animals were killed 7 weeks after inoculation (**f**) Progression of tumor size after inoculation and Dox treatment was plotted. Tumor size is shown as the mean±S.E.M. (*n*=5; *P<0.05). Representative photographs are shown for mice cohort and tumors arising in the same mice

We next questioned whether TIA1 or TIAR conditional expression could alter the growth kinetics of both established and nascent tumors. Thus, control/GFP and TIA1- or TIAR-expressing cells were injected into the right and left hind leg, respectively, of nude mice and doxycycline (Dox) was introduced into the drinking water 5 weeks later (Figure 6a). Tumor size was measured before and following Dox-induced expression of TIA1 or TIAR proteins. Compared with tumors formed by control cells, a significant and reproducible decrease in tumor size was noted in TIA1 (four out of six (67%)) and TIAR (four out of five (80%)) cells, although tumor size was more heterogeneous from TIA1-expressing cells (Figures 6b and c). Immunoblotting confirmed the expression of GFP-TIA1 and GFP-TIAR in tumors (Figures 6b and c).

Formalin-fixed, paraffin-embedded tumors were stained with hematoxylin and eosin (HE) or analyzed by immunohistochemistry for MIB1 (to evaluate proliferation), caspase-3 (for apoptosis) or BCL-2 (for anti-apoptosis). The histological features of tumors from all groups indicated poorly differentiated carcinomas (Figure 6d). Tumor cells were atypical, polygonal, epithelial in appearance, poorly differentiated with pleomorphic nuclei with granular chromatin and evident nucleolus, with little vascularization, and variable degrees of mitotic and apoptotic bodies. Epithelial cells of the control tumors were larger than corresponding cells found in TIA1 or TIAR tumors. Immunohistochemistry analysis revealed that control tumors were highly proliferative, with a cell proliferation index determined by the expression of Ki67 (MIB1 for paraffin) significantly higher in the control group, with a mean expression of 20% compared with TIA1 and TIAR groups (5% Ki67 expression, Figure 6d). Further, cells with ectopic TIA1 and TIAR expression exhibited greater apoptosis and necrosis than control cells, as shown by expression of caspase-3 and BCL-2 (Figure 6d). Accordingly, the number of cells expressing caspase-3 in control tumor sections was 10% compared with 25 and 30% found in TIA1 and TIAR groups, respectively (Figure 6d). Also, BCL-2 protein expression was found in 30% of the control group and in only 10% of cells in TIA1 and TIAR groups (Figure 6d).

As the ectopic expression of TIAR was more efficient and reproducible than TIA1 expression to drive tumor size reduction, we tested the capacity of TIAR expression to delay the growth of nascent tumors (Figure 6e). Surprisingly, the expression of TIAR 1 week after cell injection caused significant tumor reduction (five of five (100%)), and even abolition (four of five (80%); Figure 6f), suggesting that TIAR functions as a tumor suppressor gene in nascent tumors. Collectively, these findings propose that increased TIA1 or TIAR expression reduces *in vivo* tumorigenicity of FT293 cells.

### TIA proteins are downregulated in human lung squamous cell carcinomas

**Figure 7:**
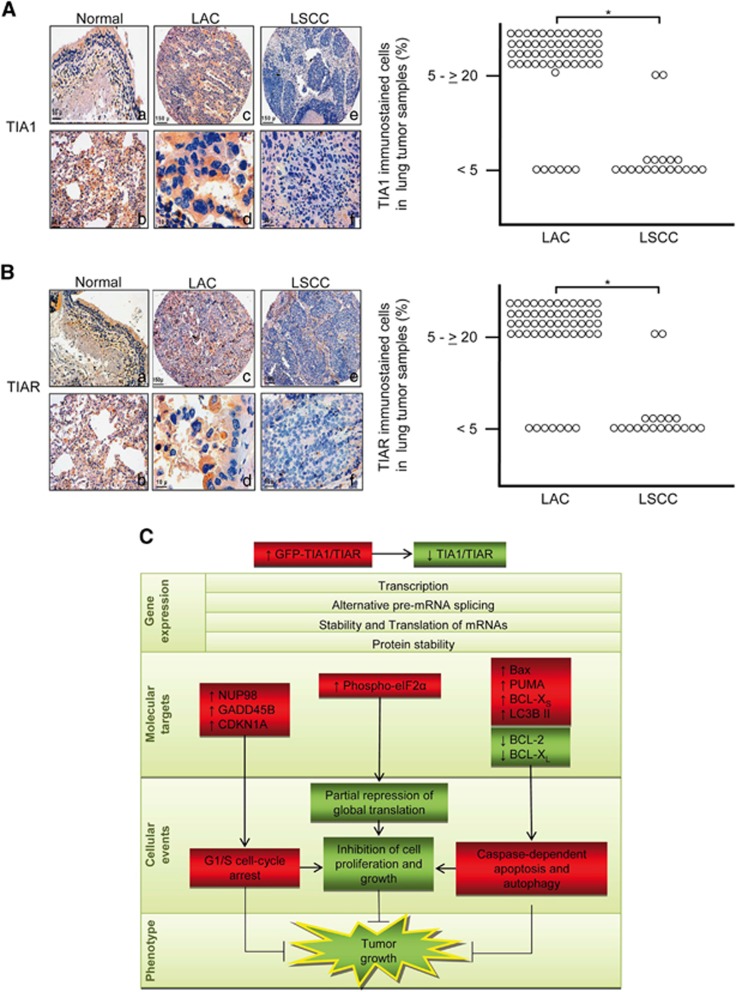
Downregulation of TIA proteins in human LSCC. (**A** and **B**) Quantification of relative expression levels of TIA1 and TIAR proteins in normal and tumor biopsies of lung cancer patients. Reduced lung TIA1 and TIAR immunoreactivity in LSCC tumors compared with non-tumor tissues (Normal) and LAC. Lung sections of patients were immunohistochemically stained with anti-TIA1 (**a**) or anti-TIAR (**b**) antibodies. Representative images of non-tumor (Normal) and lung tumor (LAC and LSCC) tissues are shown. Scale bars represent: 50 *μ*m (**a** and **b**), 150 *μ*m (**c** and **e**), 10 *μ*m (**d**) and 50 *μ*m (**f**), in each TIA1 (**a**) and TIAR (**b**) panels, respectively. Diagrams show values of the relatives percentages of TIA1 (**a**) or TIAR (**b**) immnunohistochemical staining cells in LAC and LSCC samples. Lung samples were provided by 74 lung cancer patients: 55 LAC and 19 LSCC. Statistical significance was determined from nonparametric testing (Mann–Whitney *U*-test, *P<0.0001) (**C**) A working model summarizing the molecular and cellular events linked to the ectopic expression of TIA proteins

We previously described that TIA1 and/or TIAR protein expression is decreased in a subset of human epithelial tumors.^21^ To evaluate a possible role for TIA proteins in human tumorigenesis, we analyzed TIA1 and TIAR expression in a cohort of patients with non-small-cell lung carcinoma (NSCLC). We tested 74 carcinomas (55 lung adenocarcinomas (LAC) and 19 lung squamous cell carcinomas (LSCC)) by immunohistochemistry (Supplementary Figure S9). A staining pattern for TIA1 and TIAR was detected in the cytoplasm in non-tumor lung cells (Figures 7A and B). Strikingly, strong TIA1 and TIAR staining was also detected in infiltrating inflammatory cells around the tumor regions, with a high proportion in lymphocytes, and also in adjacent non-tumor control cells (data not shown). However, weak TIA1 and TIAR staining was detected in LSCC samples (Figures 7A and B), with a significant decrease in TIA1 and TIAR immunoreactivity (*P<0.0001) in LSCC tumors compared with LAC lung tumors (Figures 7A and B). Moreover, TIA1 and TIAR expression was significantly reduced in 90% of the LSCC cases.

## Discussion

This study provides the first characterization of TIA1 and TIAR tumor suppressor activity in cancer cells. Overexpression of either TIA1 or TIAR depletes endogenous TIA proteins, illustrating an intricate regulation of TIA-associated functions.^10, 30^ This replacement of endogenous TIA proteins drives a complex process including reduction of cell proliferation, partial inhibition of global translation (in a phospho eIF2*α*-dependent manner), G1/S arrest and cell death. These cellular responses are associated with modulation of the transcriptome and/or proteome, notably linked to a subset of p53 gene targets. Together these molecular events may work in concert to suppress the growth of human tumor xenografts in a TIA-dependent manner. Further, the low expression of TIA1 and TIAR in LSCC, correlating with poor prognosis in patients, suggests that these regulators may be biomarkers and potential therapeutic targets (Figure 7C).

TIA proteins are involved in the assembly of stress granules (SG), discrete cytoplasmic inclusions containing translationally stalled initiation complexes and RNA-binding proteins that are recruited in cells subjected to environmental stress.^39^ In response to stress, TIA regulates SG formation downstream of eIF2*α* phosphorylation.^39^ Our observations show that sustained TIA expression in FT293 cells is not accompanied by massive formation of SG, as can be observed in response to the stress inducer, arsenite. However, a moderate downregulation of global protein synthesis together with phosphorylation of the eIF2*α* subunit is detected, which probably contributes to translational repression. This observation is consistent with results in mouse embryonic fibroblasts lacking TIA1 that exhibit an impaired ability to form SG, although they reveal normal eIF2*α* phosphorylation in response to arsenite.^39^ These results suggest the existence of an adaptive response involving a cytoprotective effect by 'non-canonical SG' to promote survival of stressed cells by contributing to the reprogramming of protein expression and by blocking senescence and/or pro-apoptotic signaling cascades.^39^

The tumor protein 53 responds to numerous stresses, including DNA damage, by modulating gene expression leading to enhanced DNA repair, control of cell cycle and cell death and maintenance of cellular homeostasis.^33, 34, 35, 36, 37, 38^ During stress, the transcriptional activity of p53 is regulated through multiple posttranslational modifications.^34, 38^ Only a subset of p53 target genes are known, with assessments suggesting their number to be close to 2000.^38^ These include cell cycle regulators at the G1/S phase (including NUP98, GADD45B and CDKN1A) and apoptotic inducers (including BAX and PUMA), which were upregulated in our study. These genes are key regulatory components in several signaling pathways that drive complex biological responses and can function cooperatively to inhibit cell growth and/or promote cell death (Figure 7C).^38^

CDKN1A is required for proper cell cycle progression or arrest and has a role in cell death, DNA repair, senescence and aging. CDKN1A is an important mediator to delay transit from G1 to S and/or from G2 to M, thus preventing the effects of DNA damage on gene function. CDKN1A can also regulate DNA replication within S phase.^33, 34, 35, 36, 37^ Although transcriptional control is accepted to be the initial control point for CDKN1A expression, growing evidence suggests that posttranscriptional (transport, stability and translation of mRNA) and posttranslational (protein stability and activity) regulation have a critical role in CDKN1A expression and activity.^35, 36, 37, 38^ For example, NUP98 regulates CDKN1A mRNA stability and could function as a tumor suppressor gene mediating p53 pathway signaling responses.^35^ Moreover, NUP98-interacting genes are enriched in gene regulatory events that are directly linked to the control of cell cycle.^40^ Here we showed that TIA expression induced CDKN1A accumulation without apparent major effects on protein expression levels of p53. Given that moderate changes in the steady-state levels of CDKN1A mRNA were detected, our observations suggest that TIA expression, through NUP98, could be modulating the mRNA stability/translation and/or protein stability/activity of CDKN1A.

Several p53 signaling pathway-related genes identified in this study contain multiple binding sequences of TIA proteins located across the full-length pre-mRNAs by using the TIA-iCLIP database in HeLa cells.^11^ These binding sites occur at high frequency on the last exons of these pre-mRNAs and particularly on the sequences located at the 3'-UTR of the mature mRNAs. It is therefore reasonable to assume that many of these mRNAs may be regulated by TIA proteins. Indeed, our findings show that NUP98»GADD45B=BAX=CDKN1A mRNAs can be direct TIA targets and steady-state levels of their mRNAs could be modulated by these regulators (Supplementary Figure S7). Further, our observations are also consistent with a dynamic remodeling of ribonucleoprotein complexes in response to environmental stress, involving TIA1 or TIAR expression. Notwithstanding recent studies establishing a functional intersection of RNA processing with DNA repair,^41, 42^ very few RNA processing factors have been examined for their association with the DNA damage response. Our findings suggest a potential role of TIA proteins in this scenario.

Human tumors are caused both by genetic and epigenetic events. The progressive acquisition of mutations in oncogenes or tumor suppressor genes might act in concert with epigenetic events, such as functional downregulation of TIA proteins, to give cells a competitive growth advantage. TIA1 and TIAR are mutated in several types of human cancers. The IntOGen-mutations platform (www.intogen.org/mutations) summarizes somatic mutations, genes and pathways involved in tumorigenesis.^43^ Analysis of this database provides support to link human cancers with somatic mutations in TIA1 and/or TIAR/TIAL1 (Supplementary Figure S10). In human cells, TIA proteins regulate the transcription, splicing, stability and/or translation of many genes associated with the hallmarks of cancer.^21^ Thus, the aberrant expression of TIA proteins could facilitate the acquisition of oncogenic phenotypes.^21^ Accordingly, mice with disruption in the *tiar* gene develop ovarian sex cord stromal tumors.^23^ Further, TIA proteins regulate alternative splicing of WT1^44^ and NF1^45^ tumor suppressor genes, implicated in development of childhood kidney cancer, neurofibromatosis and juvenile leukemia, respectively. Several well-established oncogenes, including FGFR2, C-MYC, PTGS2 and HIF1A, among others, are TIA-regulated gene targets at the transcriptional and/or posttranscriptional levels.^7, 11, 12, 13, 14, 15, 16, 17, 18, 20, 21^ Further, TIA1 expression in tumors strongly correlates with responsiveness to immunotherapy in melanoma and sarcoma patients.^46, 47, 48, 49^

Lung cancer is the most common cause of cancer-related mortality worldwide, with a 5-year-survival rate of 16%.^50^ Lung cancers are divided into two major histological classes: small-cell lung cancer (SCLC) and NSCLC, which represents 80% of all lung cancer cases. NSCLC is further divided into adenocarcinoma (LAC), squamous cell carcinoma (LSCC), and large cell carcinoma (LCLC). Recent therapies targeting NSCLC subtypes have resulted in encouraging new treatments for LAC, driven by EGFR or EML4-ALK mutations. However, there are relatively few treatment options for LSCC, a carcinoma that represents 30% of all lung cancer diagnoses and is characterized by poor therapeutic response, a high relapse rate and poor prognosis. Thus, there is a need to better understand molecular mechanisms that drive LSCC and to translate this knowledge to superior intervention strategies. Our results suggest that TIA proteins could be good biomarkers of LSCC.

The regulatory impact of TIA proteins is similar to well-documented genes with features of cellular gatekeepers. TIA1 and TIAR gene disruption or overexpression in murine models provokes early embryonic lethality,^16, 23, 24, 25^ similar to other tumor suppressor genes.^51, 52^ Equally, loss-of-function of these tumor suppressors in transformed cells improves cell and tumor growth. Conversely, gain-of-function of these regulators triggers cell cycle arrest and cell death. Thus, data from knockout murine models^16, 23^ and human tumor xenografts using different tumor cell lines, such as HeLa^21^ (Supplementary Figure S11), A549,^20^ LS174t,^53^ HEK293 (this study), together with expression data from human tumors (ref. 21 and this study), and the identification of somatic mutations in human tumor samples from different origins,^43^ strongly suggest that it may advantageous for some human tumors to reduce TIA1 and/or TIAR activity/expression, since these effectors could act under specific environmental circumstances as tumor suppressors and barrier, that is, as cellular gatekeepers, to cancer progression.

## Materials and Methods

### Cell lines

FT293 cell lines expressing GFP-tagged proteins were generated using the Flp-In T-REx System (Invitrogen, Carlsbad, CA, USA). For protein labeling, FT293 cells were incubated with methionine-cysteine free DMEM supplemented with 5 *μ*l Easy Tag EXPRESS [^35^S] Protein Labeling mix (Perkin Elmer, Waltham, MA, USA) for 30 min. Transient RNA interference of human CDKN1A mRNA was carried out with siRNA duplexes (SR300740, Origene, Rockville, MD, USA).

### Protein, western blot and immunofluorescence analysis

FT293 cell lines were processed for western blot and immunofluorescence analysis as reported.^18, 21, 25^

### Proliferation, cell cycle and cell death analysis

Cell proliferation, cell cycle and cell death rates were quantified as described.^18, 21, 25^ For caspase inhibition, cells were incubated with 50 *μ*M pan-caspase inhibitor benzyloxycarbonyl-Val-Ala-Asp (OMe) fluoromethylketone (Z-VAD-FMK, Bachem, Bubendorf, Switzerland).

### RNA isolation, transcriptome and qRT-PCR (qPCR) analysis

RNA purification, microarray (Agilent, Santa Clara, CA, USA; SurePrint G3 Human Gene Expression 8x60K v2, version G4851B) and qPCR analysis were carried out as described.^25^ Microarray data generated in this work have been deposited in the NCBI Gene Expression Omnibus database (http://www.ncbi.nlm.nih.gov/geo/info/linking.html) and are accessible through the GEO Series accession number GSE51500. The following link has been created to allow review of record GSE51500 while it remains in private status: http://www.ncbi.nlm.nih.gov/geo/query/acc.cgi?token=wzeheikqbpsxbaz&acc=GSE51500. GO and KEGG database analyses were conducted using software programmes provided by GenCodis3 (http://genecodis.cnb.csic.es).^25^

### Polysome analysis using sucrose gradients

Subconfluent FT293 cell lines were processed as reported.^26^

### Immunoprecipitation (IP) and quantitative RT-PCR (IP-qPCR) analysis

IP-QPCR assays were performed using GFP-Trap M reagent (Chromotek, Planegg, Germany). The antibody–antigen complexes were processed for western blot and qPCR analysis.

### mRNA and protein stability assays

FT293 cell lines were grown in the presence of either 5 *μ*g/ml of Act D (Sigma, St. Louis, MO, USA) or 50 *μ*g/ml of CHX (Sigma), and total RNAs or proteins were isolated at various time intervals. The mRNA and protein expression levels were measured by qPCR and immunoblotting, respectively.

### Xenograft tumor development in nude mice, HE staining and immunohistochemistry

FT293 cell lines (5 × 10^6^ cells per site in 200 *μ*l) were injected s.c. into each rear flank in nude mice (Harlam Ibérica nu/nu). Tumor growth was monitored weekly in mice before and after addition of Dox (2 mg/ml) to the drinking water. Five to six mice were used per experimental condition. Formalin (4%)-fixed, paraffin-embedded tumor samples were sectioned and stained with HE as described.^21^ Immunohistochemical analysis was carried out as reported.^21^ All experiments were performed with 8–12-week-old female mice and conducted according to the guidelines of the Committee on Animal Experimentation of the Centro de Biología Molecular Severo Ochoa and Consejo Superior de Investigaciones Científicas. Quantification of the relative expression of TIA1 and TIAR proteins in normal and tumor biopsies of lung cancer patients was carried out from normal (Control) and tumor biopsies of each lung cancer patient (55 LAC and 19 lung squamous cell cancer (LSCC)).

### Bioethics

Patients' medical records were reviewed and identifiers coded to protect patient confidentiality. Anonymized and coded normal and tumor samples supplied by the Banco de Tejidos y Tumores of the Hospital Universitario de Salamanca were provided with informed consent from the patients and obtained after approval of the corresponding Institutional Review Board.

### Statistical analysis

All data were expressed as mean±S.E.M. Paired two-tailed Student's *t*-test was applied to determine statistical significance between two groups. *P*-values <0.05 were considered statistically significant. Statistical significance of immunohistochemical analysis from human lung tumor samples was determined from nonparametric testing using Mann–Whitney U-test.

## Abbreviations

BAX: BCL-2-associated X protein
BCL-2: B-cell CLL/lymphoma 2
CDKN1A: cyclin-dependent kinase inhibitor 1A
C-MYC: Myc proto-oncogene protein
DOX: doxycycline
GADD45B: growth arrest and DNA-damage-inducible, beta
GAPDH: glyceraldehyde-3-phosphate dehydrogenase
GO: gene ontology
HuR: Hu antigen R
*γ*H2AX: gamma-H2A histone family, member X
iCLIP: *in vitro* ultraviolet-crosslinking and immunoprecipitation assay
KEGG: Kyoto Encyclopedia of Genes and Genomes
LAC: lung adenocarcinoma
LC3B: microtubule-associated protein 1 light chain 3 beta
LSCC: lung squamous cell carcinoma
NSCLC: non-small-cell lung cancer
NUP98: nucleoporin 98 kDa
SG: stress granules
TIA1: T-cell intracellular antigen 1
TP53: tumor protein 53
