# Supplementary material for: T-cell intracellular antigens function as tumor suppressor genes
Source: Cell Death Dis. 2015 Mar 5;6(3):e1669–. doi: 10.1038/cddis.2015.43 (PMC4385921; doi:10.1038/cddis.2015.43)

T-cell intracellular antigens function as tumor suppressor genes

Carmen Sánchez-Jiménez[1], María Dolores Ludeña[2] and José M. Izquierdo[1, 3]

[1]Centro de Biología Molecular Severo Ochoa
Consejo Superior de Investigaciones Científicas
Universidad Autónoma de Madrid (CSIC/UAM)
C/ Nicolás Cabrera 1. 28049 Madrid. Spain

[2]Universidad de Salamanca-Hospital Universitario de Salamanca
Facultad de Medicina. Departamento de Biología Celular y Patología
C/ Paseo de San Vicente 58-182. 37007 Salamanca. Spain

Running title: TIA proteins are cellular gatekeepers

Keywords: TIA1, TIAR, cell-cycle, cell death, tumorigenesis

Competing interests: The authors declare that they have no competing interests

Financial support: The Spanish Ministry of Innovation and Science through FEDER funds (BFU2008-00354 and BFU2011-29653 to JMI) and Junta de Castilla y León (GRS 600/A/11 to MDL). The CBMSO receives an institutional grant from Fundación Ramón Areces.

[3]**Correspondence to:**
José M. Izquierdo
Email: jmizquierdo@cbm.csic.es

Supplementary Figures/Additional files
The following additional data are available with the online version of this paper:

Supplementary Figure S1: Additional file 1. Time-course of the GFP-tagged proteins expressed from FT293 cell lines. Exponentially growing 293 Flp-In T-Rex cells expressing GFP, TIA1, TIAR, TIA1ΔQ, TIARΔQ and HuR proteins were fixed, stained with To-Pro-3 and visualized by fluorescence microscopy for times indicated upon tetracycline (Tet) induction. In all cell lines used in our study upon Tet treatment for 48 hours, we have verified the formation of stress granules with stress oxidative agent sodium arsenite for 1 hour at 0.5 mM. The scale bar shows 20 μm.

Supplementary Figure S2: Additional file 2. Expression of TIA1 and TIAR induces splicing switch of human TIA1 and TIAR pre-mRNAs. (**A**) Down-regulation of endogenous TIA proteins by over-expression of ectopic TIA1 or TIAR proteins. Western blot analysis of cells extracts from FT293 cells expressing GFP, HuR, TIA1, TIAR, TIA1ΔQ and TIARΔQ upon tetracycline (Tet) induction for 3 days. The blots were incubated with the antibodies indicated. Molecular weight markers and the identities of protein bands are shown. (**B**) TIAR overexpression induces splicing of human TIA1 gene novel alternative exons. Schematic representation of the human TIA1 gene. Constitutive exons are represented by boxes. The major mRNA isoforms of TIA1 are shown identified as TIA1a and TIA1b isoforms including or not the exon 5. Positions of primers used in the RT-PCR analysis are indicated by arrows. For each primer pair, the RT-PCR products are shown below the gene map. RT-semiquantitative (sq)PCR analysis were performed on RNAs purified from cell lines indicated. The novel spliced products are identified with one, two or three asterisks, respectively, and a scheme with the composition of exons determined by DNA sequencing. (**C**) TIA1 overexpression induces splicing of human TIAR gene novel alternative exons. Schematic representation of the human TIAR gene. Constitutive exons are represented by boxes. The major mRNA isoforms of TIAR are shown identified as TIARa and TIARb isoforms involving the selection of alternative 3' splice sites on exon 3. Positions of primers used in the RT-PCR analysis are indicated by arrows. For each primer pair, the RT-PCR products are shown below the gene map. RT-PCR analysis were performed on RNAs purified from cell lines indicated. The novel spliced products are identified with one, two or three asterisks, respectively, and a scheme with the composition of exons determined by DNA sequencing. Inclusion of exon 5a leads to a frameshift and premature termination. Exons 6a, 8a and 11a contain in frame stop codons. (**D**) RNA map of TIA proteins on human TIA1 and TIAL1 pre-mRNAs in HeLa cells identified by *in vivo* UV-crosslinking and immunoprecipitation (iCLIP) analysis. The RNA maps were adapted using the TIA-iCLIP database provided by Jernej Ule's laboratory. The bar graphs show the number of cDNAs that identified each crosslinking site. The localization of target genes on human chromosomes and the exon and intron positions of the human pre-mRNAs are shown. The following gene names are used: TIA1, T-cell intracellular antigen 1 and TIAL1/TIAR, TIA1 like/related gene.

Supplementary Figure S3: Additional file 3. List of primer pair sequences used in quantitative (Q)RT-PCR and semiquantitative RT-PCR analysis.

Supplementary Figure S4: Additional file 4. Senescence analysis with SA-β-gal staining. FT293 cells expressing GFP, HuR, TIA1 or TIAR were subjected to SA-β-gal

staining using senescence cells histochemical staining kit (Sigma) according to manufacturer's instructions.

Supplementary Figure S5: Additional file 5. Summary of differentially expressed genes in FT293 cells expressing GFP, HuR, TIA1 or TIAR proteins.

Supplementary Figure S6: Additional file 6. List of clustered genes by GO and KEGG database analysis from microarray-predicted genes from above cell lines.

Supplementary Figure S7: Additional file 7. Immunoprecipitation of RNA-protein (RNP) complexes associated to GFP-tagged HuR, TIA1 and TIAR proteins. (**A**) Cleared extracts from GFP, GFP-HuR, GFP-TIA1 and GFP-TIAR-expressed FT293 cells were incubated with GFP-Trap^R M reagent (Chromotek), according to the manufacture's protocol. Immunoprecipitated and washed pellets containing antibody-antigen complexes were analyzed by western blotting using anti-GFP antibody. RNAs associated with the antibody-antigen complexes were eluted, deproteinized, precipitated and treated with RNA-free DNase, and finally analyzed by QPCR using appropriated primer pairs (Supplementary Figure S3). In all cases, the represented values are means $\pm$ SEM (n = 2; * $P < 0.05$; **$P < 0.01$; ***$P < 0.001$). (**B**) RNA map of TIA proteins on human gene targets identified in our study by *in vivo* UV-crosslinking and immunoprecipitation (iCLIP) analysis. Crosslinking sites of TIA1 and TIAR on these human genes in HeLa cells. The bar graph shows the number of cDNAs that identifies each crosslinking site. The following gene names are used: GAPDH, glyceraldehyde-3-phosphate dehydrogenase; GADD45B, growth arrest and DNA-damage-inducible beta; NUP98, nucleoporin 98 kDa; BAX, BCL2-associated X protein; CDKN1A, cyclin-dependent kinase inhibitor 1 A (p21, Cip1).

Supplementary Figure S8: Additional file 8. Transient depletion of CDKN1A in FT293 cells doesn't retrieve the cell phenotype. (**A**) Molecular and cellular events associated to the TIA1 and TIAR expression in FT293 cells were partially reverted by removing ectopic TIA1 and TIAR proteins for 4 days. Gene targets and cell-cycle phases were analyzed by immunoblotting and flow cytometry, respectively. (**B**) Western blot analysis of FT293 cells expressing GFP, GFP-TIA or GFP-TIAR proteins. Extracts (10 μg) prepared 72 h after transfection with siRNAs (Origene) against control (c is an universal scrambled negative control siRNA duplex) and p21/CDKN1A (samples 1-3 are 3 unique 27-mer siRNA duplexes against human CDKN1A mRNA (SR300740)). Silencing of 70-80% of p21/CDKN1A levels were reproducibly achieved under these conditions. The blot was probed with antibodies against GFP, p21/CDKN1A, and α-tubulin proteins, as indicated. Molecular weight markers and the identities of protein bands are shown. (**C**) Analysis of cell-cycle phases from above FT293 cells (**B**) by flow cytometry after propidium iodide staining. The percentage of cells was quantified in every cell-cycle phase for each experimental condition analyzed. The represented values are means $\pm$ SEM (n = 3).

Supplementary Figure S9: Additional file 9. Summary of the clinico-pathological characteristics of the cohort of lung cancer patients studied for analysis of the expression of TIA1 and TIAR proteins. LAC, lung adenocarcinoma and LSCC, lung squamous cell carcinoma.

Supplementary Figure S10: Additional file 10. List of somatic mutations of TIA1 and TIAR genes detected across the cohort of human tumor samples. (**A** and **B**) The IntOgen-mutations platform (http://www.intogen.org/mutations/) summarizes somatic mutations, genes and pathways across tumor types involved in tumorigenesis and systematically analyzes the data across various sequencing projects. The graphics shown for TIA1 (**A**) and TIAR/TIAL1 (**B**) genes are adapted from graphics and schemes generated by this bioinformatic tool.

Supplementary Figure S11: Additional file 11. The ectopic expression of TIAR in HeLa cells represses cell proliferation and tumor growth. (**A**) Western blot analysis of HeLa cells control or expressing GFP and GFP-TIAR proteins. The blot was probed with specific antibodies against GFP and α-tubulin proteins, as indicated. Molecular mass markers and the identities of protein bands are indicated. (**B**) The ectopic expression of TIAR inhibits cell proliferation. HeLa cells were seeded in six-well plates and the total number was counted daily for 6 days. Each point represents the means ± SEM (n = 2). (**C**) The ectopic expression of TIAR retards cell growth. HeLa cells grown for 6 days were monitored by methyl thiazolyl tetrazolium (MTT) assay. The represented values were normalized and expressed relative to GFP, which was assigned an arbitrarily fixed value of 100, and are shown as means ± SEM (n = 3). (**D**) The ectopic expression of GFP-TIAR increases the percentage of apoptotic HeLa cells (identified by the sub G1-phase peak) and decreases the proportion of HeLa cells in G2/M phase. Flow cytometry after propidium iodide staining. The results are means ± SEM (n = 3). (**E**) The ectopic expression of TIAR represses anchorage-dependent growth. HeLa cells were fixed in paraformaldehyde (5%), stained with Crystal Violet (0.01%) and quantified by light microscopy. The values shown are means ± SEM (n = 17 fields). (**F-H**) The overexpression of TIAR retards tumor cell growth *in vivo*. (**F**) HeLa cells were injected s.c. into the hind legs of female nude mice. In all cases, pairs of HeLa cells expressing GFP and GFP-TIAR proteins were injected. Animals were killed 10 weeks after injection of the cells. (**G**) Representative photographs for tumors arising in the same mice. (**H**) Progression of tumor sizes after inoculation. Tumor sizes are shown as the means ± SEM (n = 4) volume in mm^3.

Additional file 1

Endogenous TIA1, TIAR and HuR proteins in 293 Flp-In T-Rex (FT293) cells

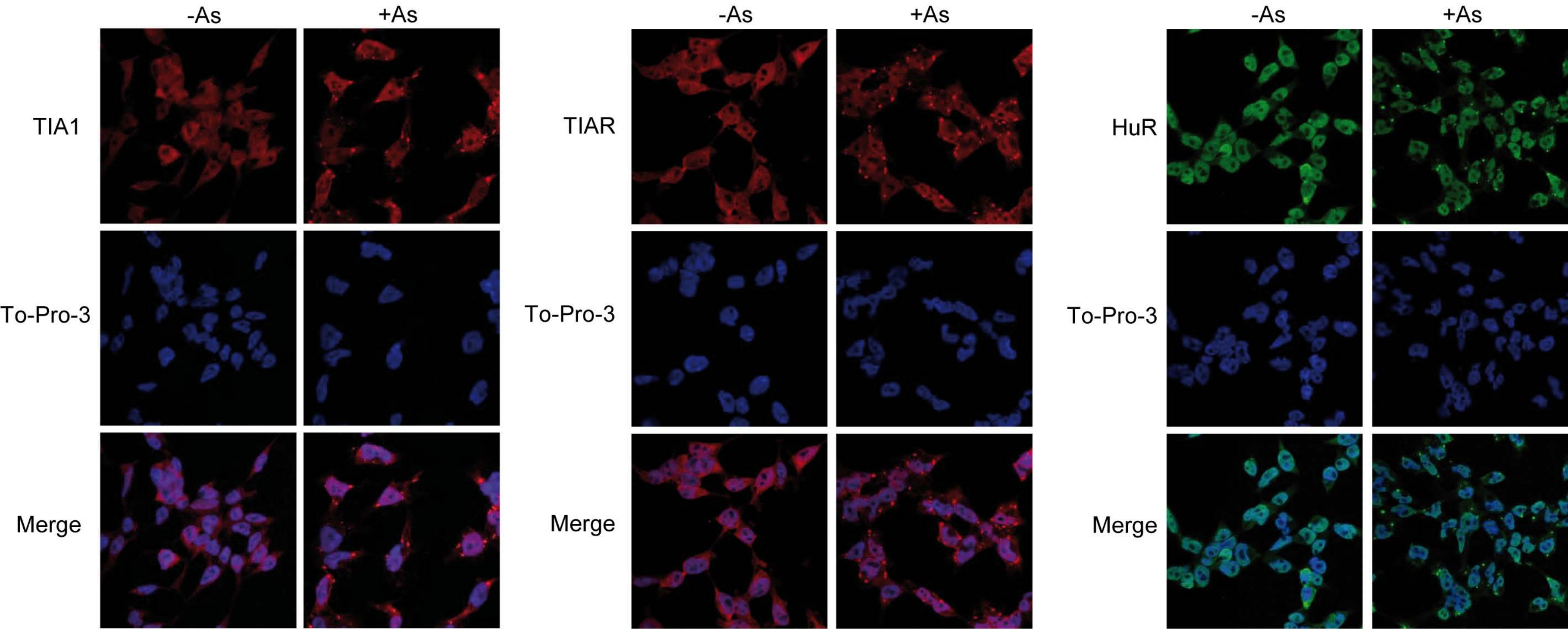

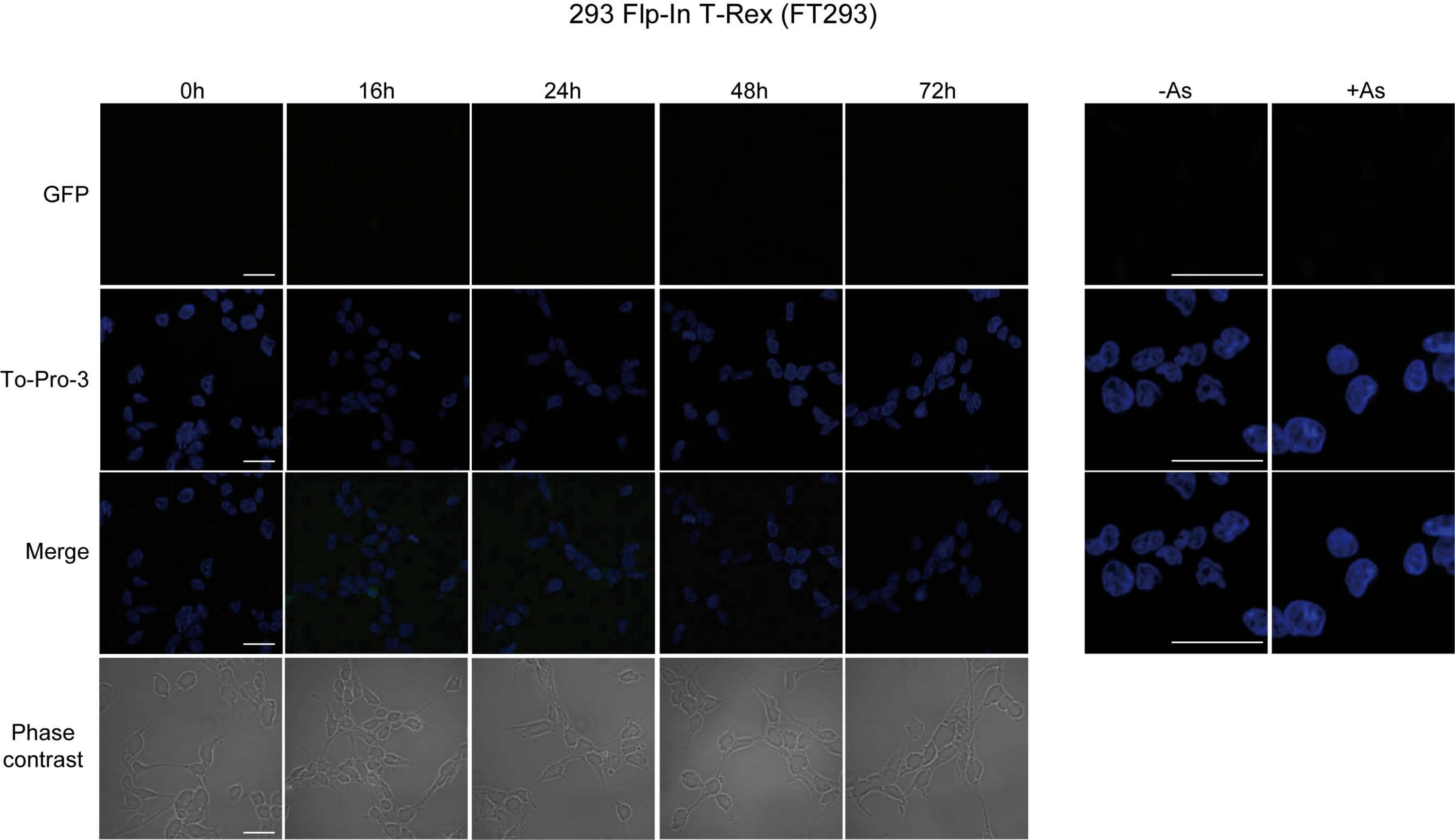

293 Flp-In T-Rex (FT293)
0h
16h
24h
48h
72h
-As
+As
GFP
To-Pro-3
Merge
Phase contrast

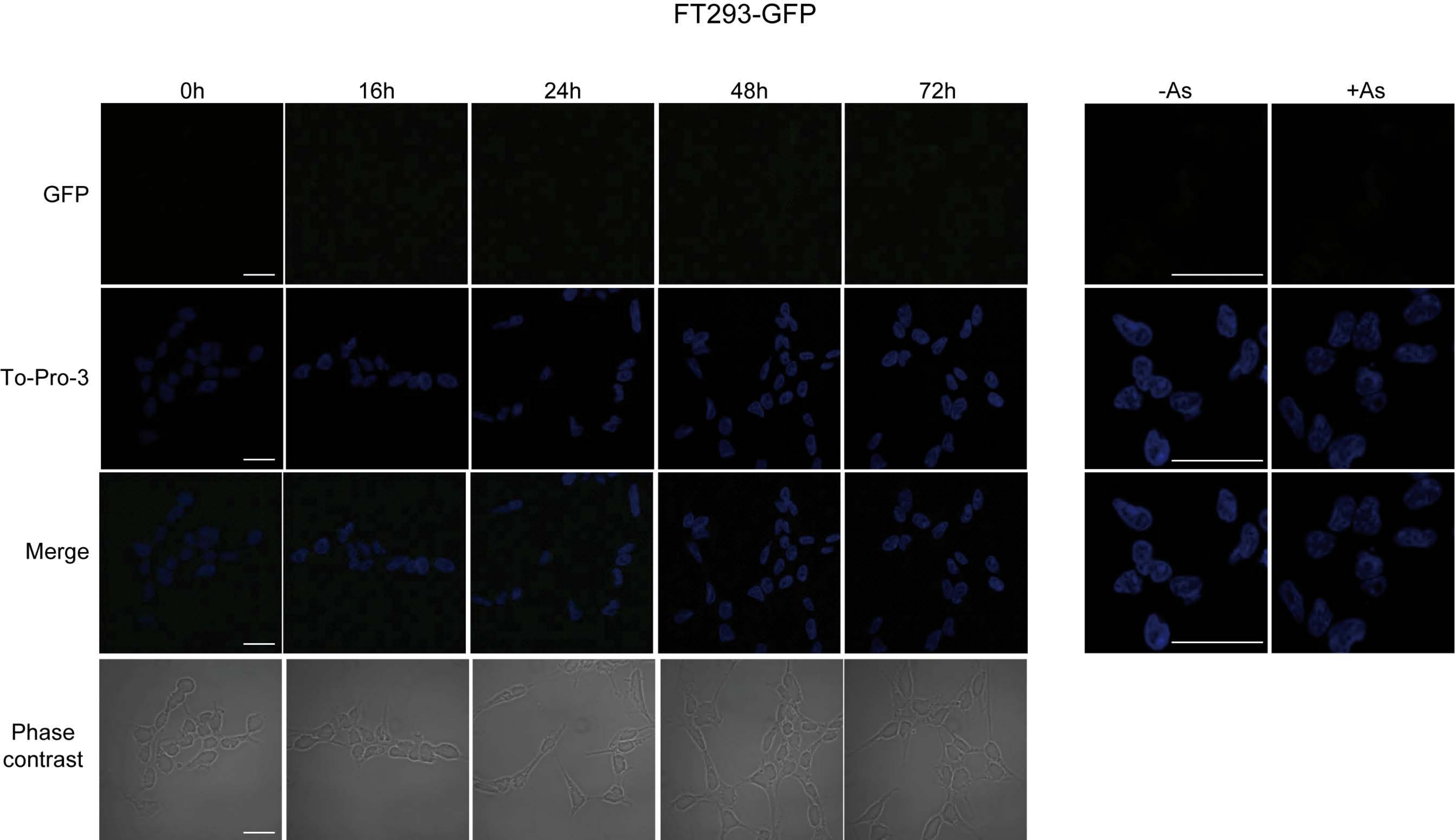

FT293-GFP
0h
16h
24h
48h
72h
-As
+As
GFP
To-Pro-3
Merge
Phase contrast

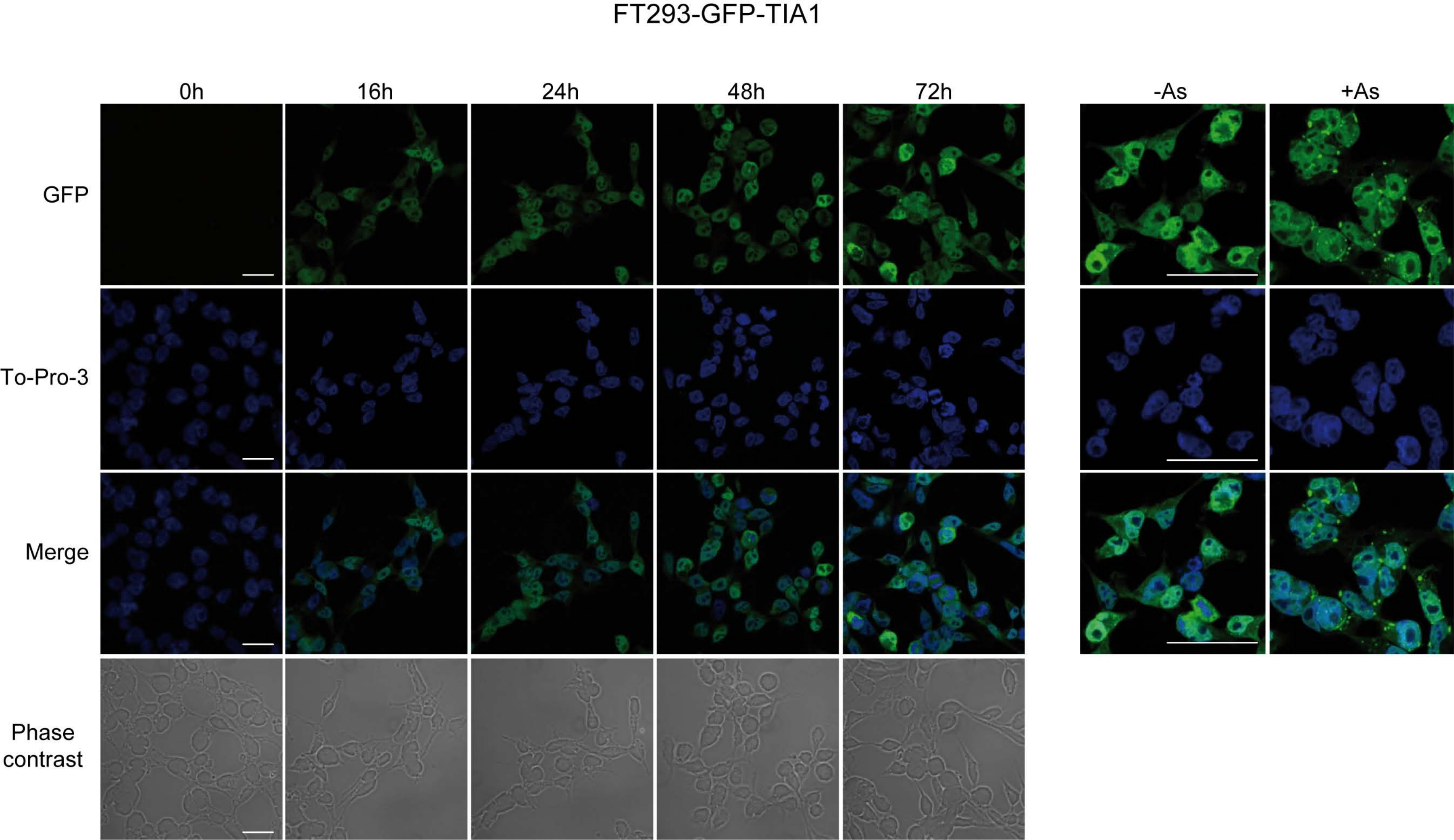
FT293-GFP-TIA1
0h
16h
24h
48h
72h
-As
+As
GFP
To-Pro-3
Merge
Phase contrast

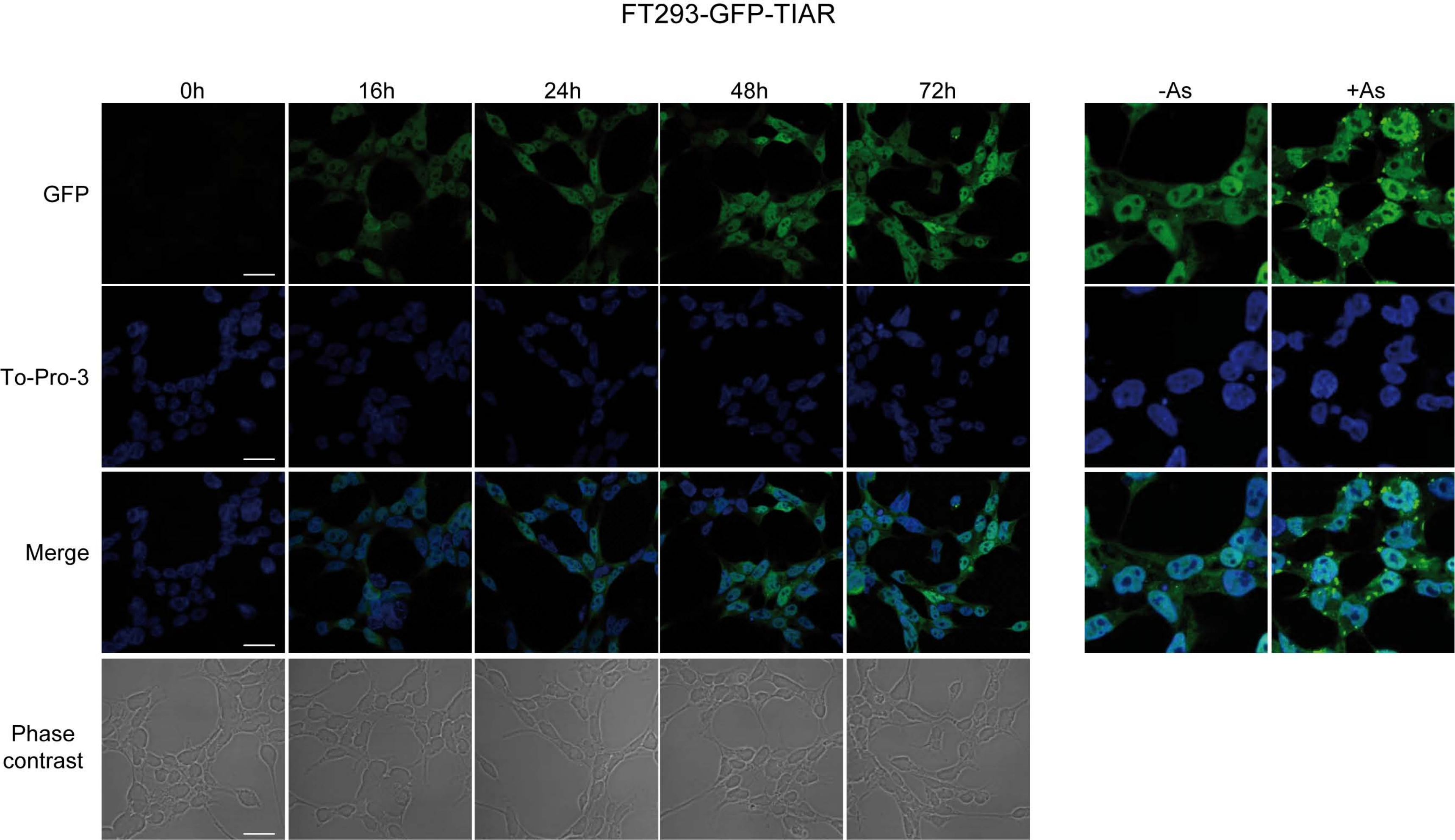
FT293-GFP-TIAR
0h
16h
24h
48h
72h
-As
+As
GFP
To-Pro-3
Merge
Phase contrast

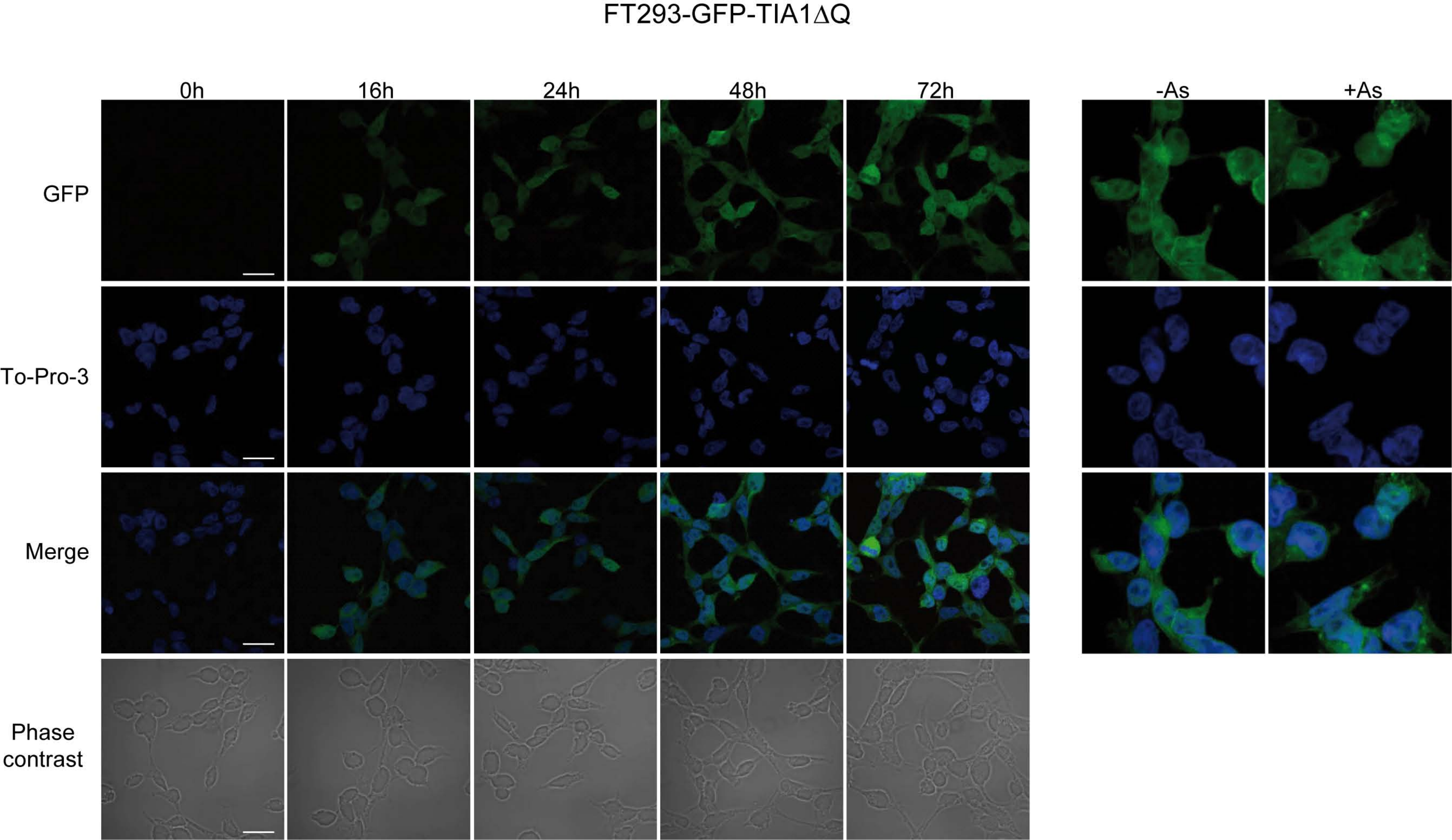
FT293-GFP-TIA1ΔQ
0h
16h
24h
48h
72h
-As
+As
GFP
To-Pro-3
Merge
Phase contrast

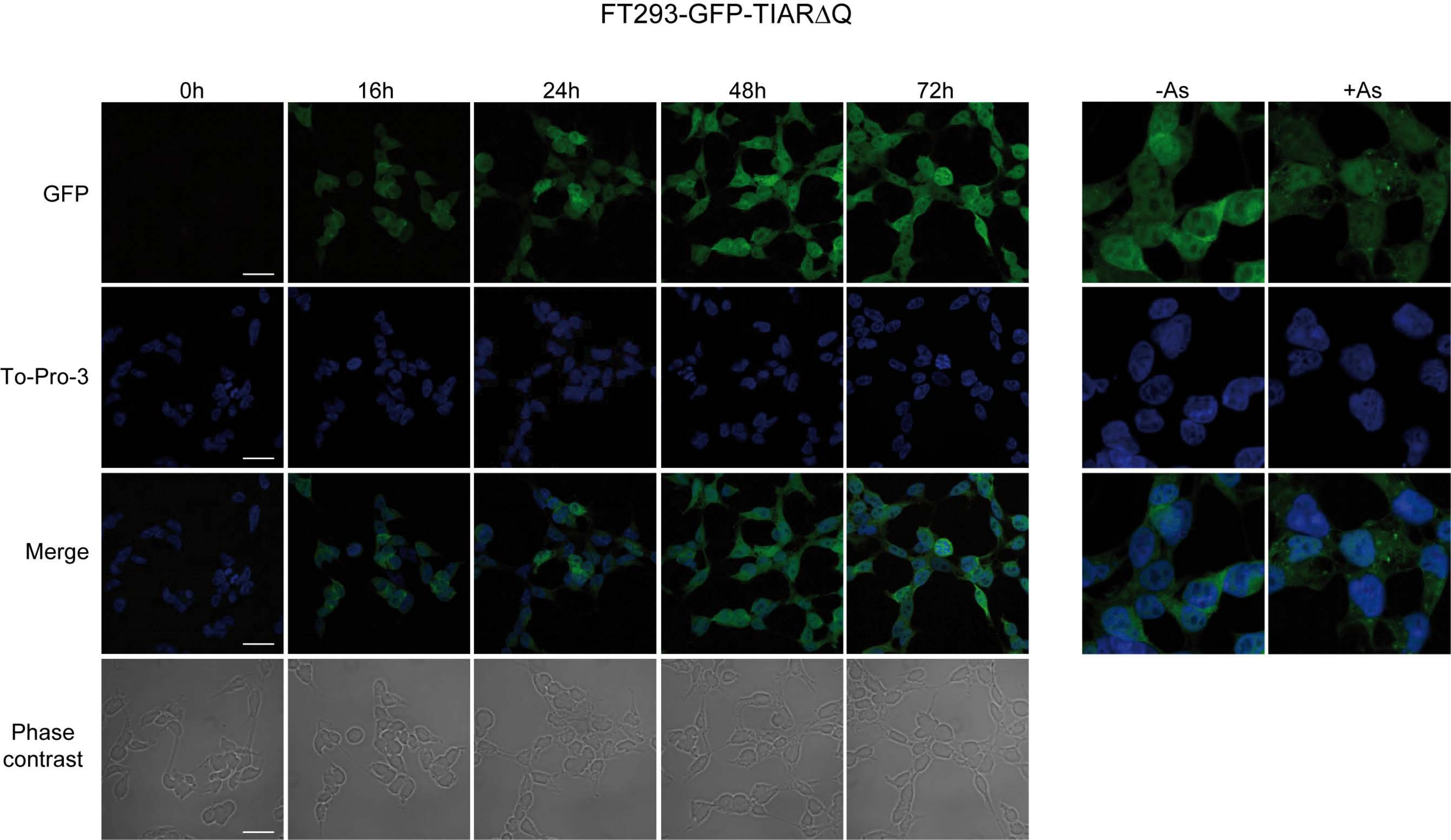
FT293-GFP-TIARΔQ
0h
16h
24h
48h
72h
-As
+As
GFP
To-Pro-3
Merge
Phase contrast

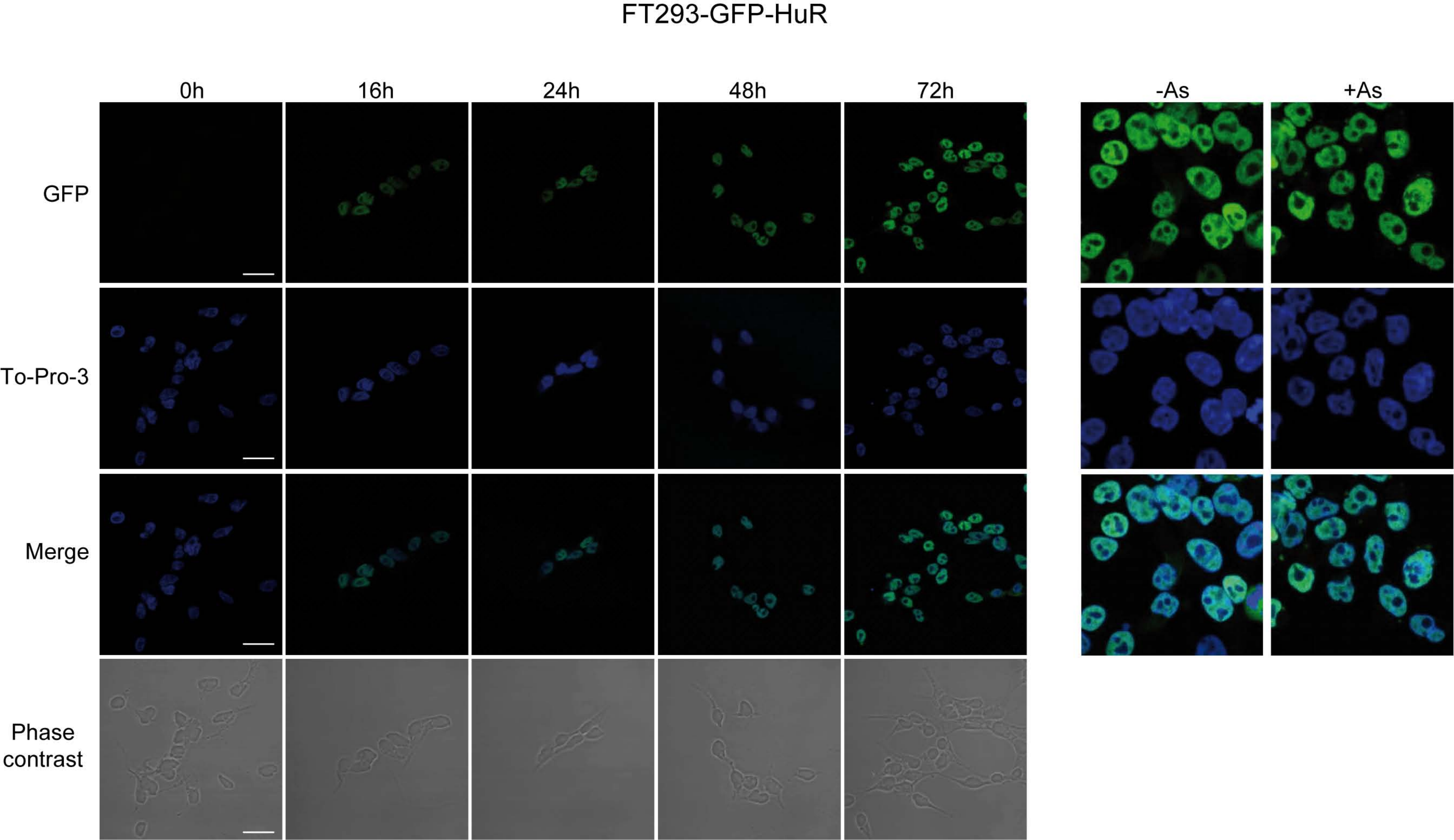

FT293-GFP-HuR
0h
16h
24h
48h
72h
-As
+As
GFP
To-Pro-3
Merge
Phase contrast

Additional file 2

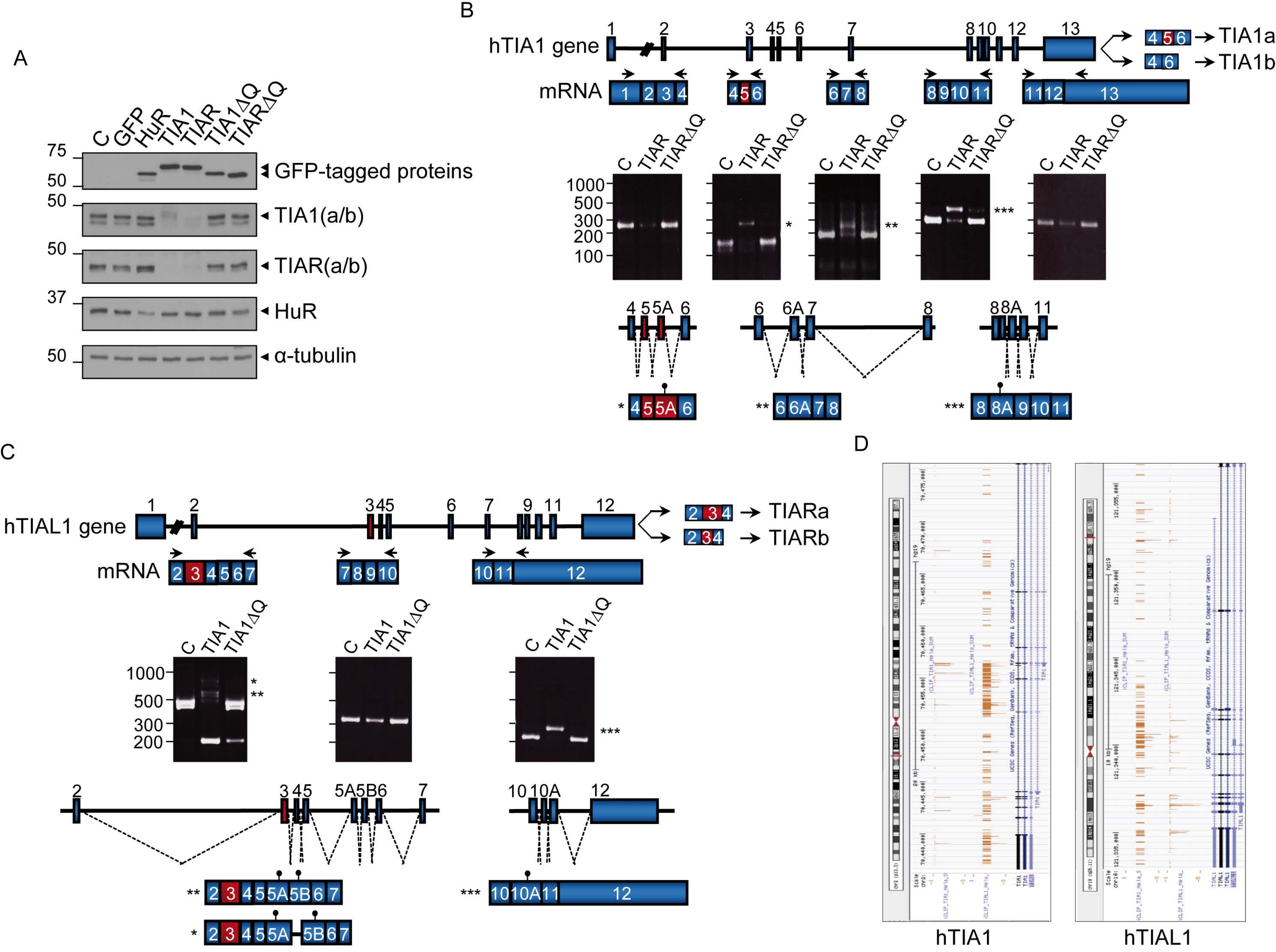

Additional file 4

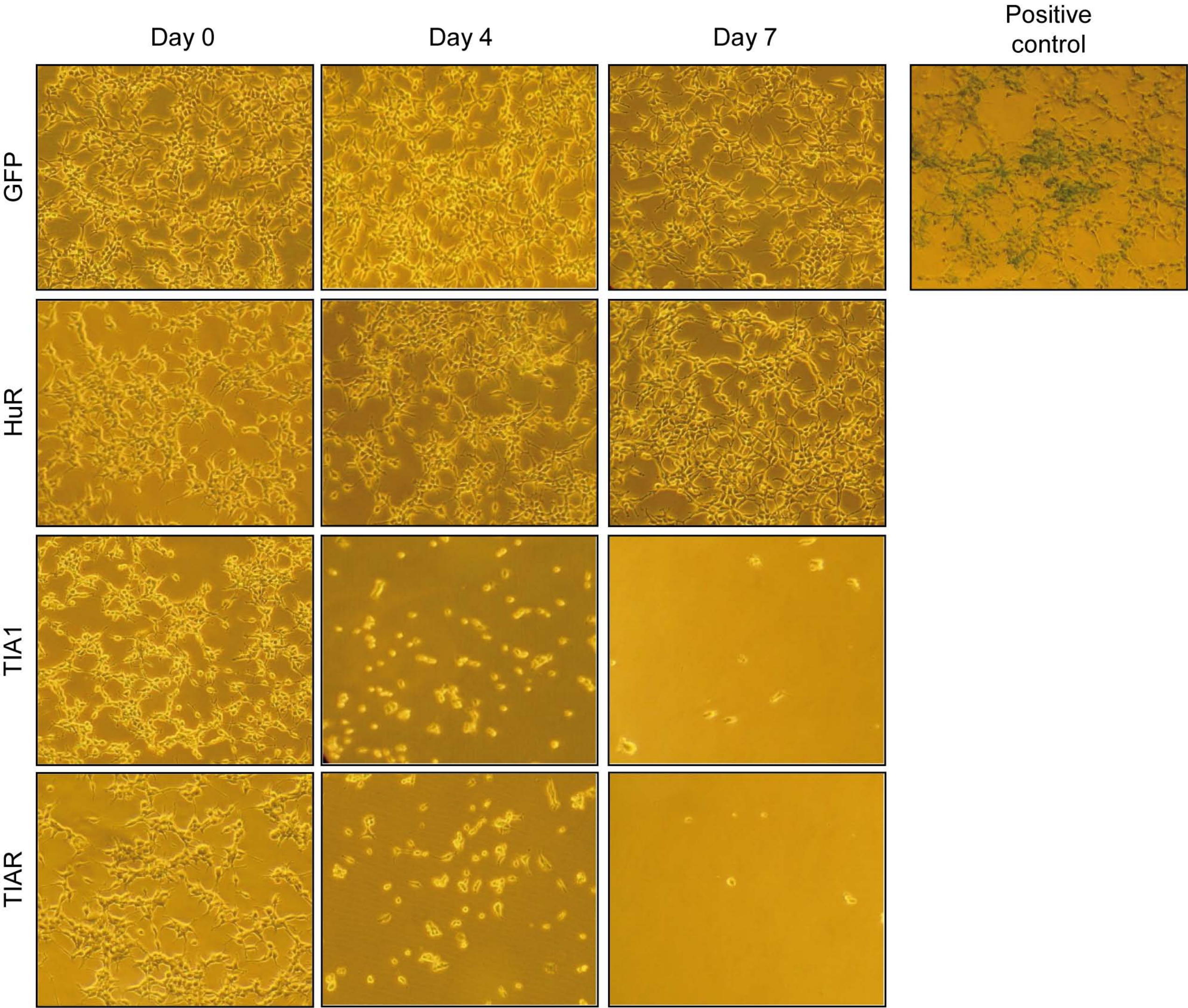

Additional file 7

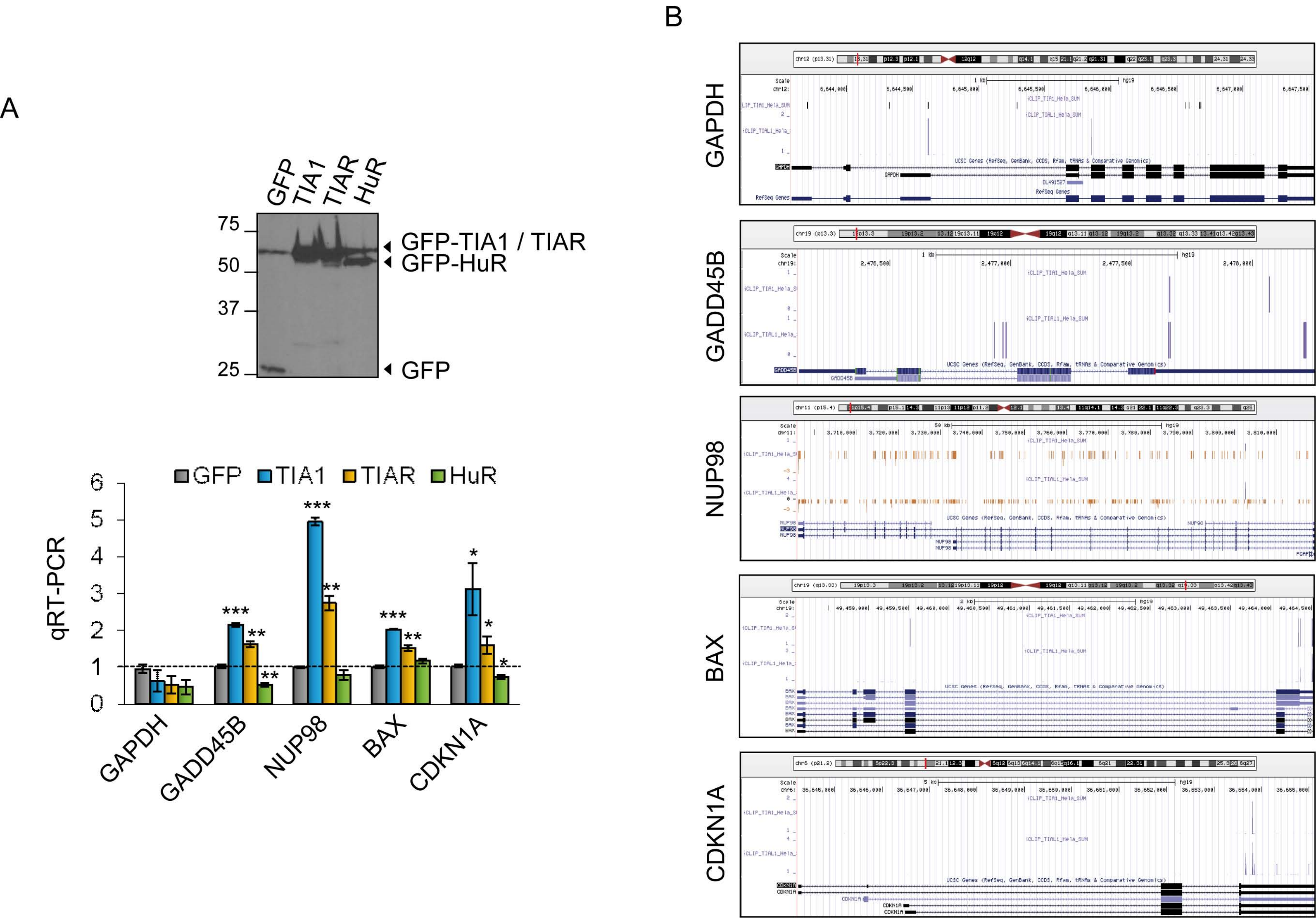

Additional file 8

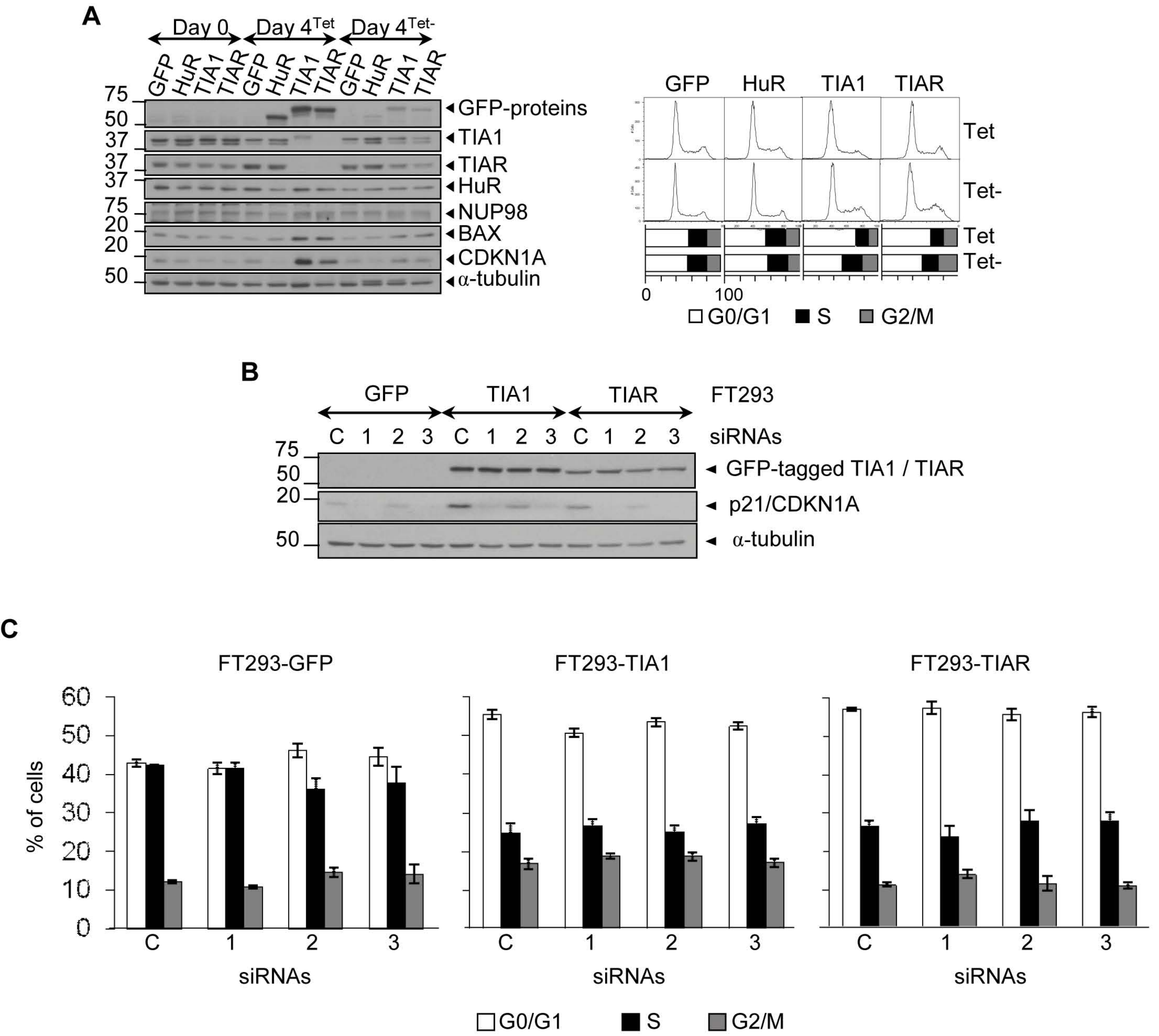

Additional file 9. Summary of the clinico-pathological characteristics of the cohort of lung cancer patients studied for analysis of the expression of TIA1 and TIAR proteins. LAC, lung adenocarcinoma and LSCC, lung squamous cell carcinoma. No. indicates the number of biopsies in each group.

**Characteristics**		**No.**
**Control**		8
**Tumor**		74
**Sex**		
	Female	14
	Male	60
**Age**		
	65	39-79
**Histology**		
	LAC	55
	LSCC	19
**Habit**		
	Smoker	60
	Non-smoker	14
**Grade**		
	IA	20
	IB	27
	IIA	3
	IIB	13
	IIIA	9
	IIIB	2

Additional file 10

A

Mutation frequency per cancer site

Mutation frequency per project

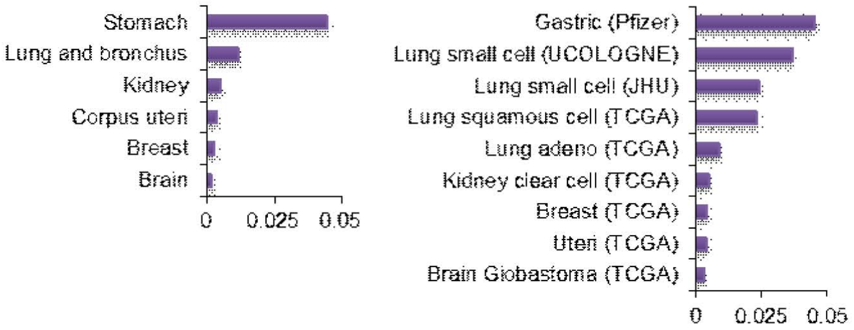

Protein change	Cancer type	Mutated samples	Impact
G315A, G325A,G97A…	Kidney	1/417	Medium
fs9, r.spl?	Breast	1/1148	Hight
H96Y, H88Y,H107Y…	Breast	1/1148	Low
C/G: D289H, D278H, D60H	Breast	1/1148	Medium
F335S, F334S, F324S…	Lung and bronchus	1/665	Low
Y48C, Y40C	Lung and bronchus	1/665	Medium
A56P, A64P	Lung and bronchus	1/665	Medium
P84R, P76R	Lung and bronchus	1/665	Low
V118=, V137=, V126=	Lung and bronchus	1/665	None
R244Q, R15Q, R233Q	Lung and bronchus	1/665	Medium
P101L, P319L, P330L…	Lung and bronchus	1/665	Low
Q330L, Q112L, Q340L…	Lung and bronchus	1/665	Low
fs 53, fs 45	Brain	1/492	Hight
G168=, G157=	Corpus uteri	1/230	None

B

Mutation frequency per cancer site

Mutation frequency per project

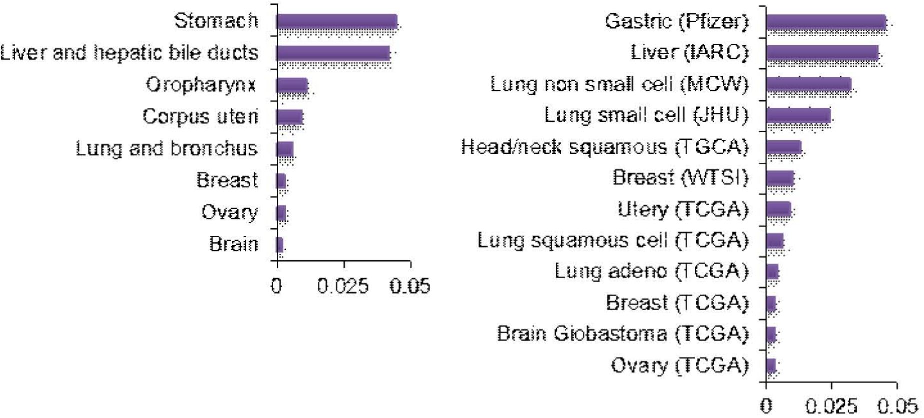

Protein change	Cancer type	Mutated samples	Impact
T109I, T70I, T126I	Ovary	1/316	Medium
D46N	Corpus uteri	1/230	None
Q31E	Breast	1/1148	Low
M109R, M232R, M249R	Breast	1/1148	Low
Q169E, Q309E, Q292E	Breast	1/1148	Low
V20=	Lung and bronchus	1/665	None
R4L, R144L, R88L	Lung and bronchus	1/665	Low
A365=, A382=, A242=	Lung and bronchus	1/665	None
N225D, N208D, N85D	Liver and hepatic bile ducts	1/24	Medium
-/ALAA 28-29, -/ALAA 67-68, -/ALAA 84,85	Oropharynx	1/375	None
r.spl?	Oropharynx	1/375	Hight
T264M, T141M, T281M	Oropharynx	1/375	Low
P194=, P317=, P334=	Oropharynx	1/375	None
fs 70, fs 126, fs 109	Brain	1/492	Hight
fs4, r.spl?, fs 43	Corpus uteri	1/230	Hight

Additional file 11

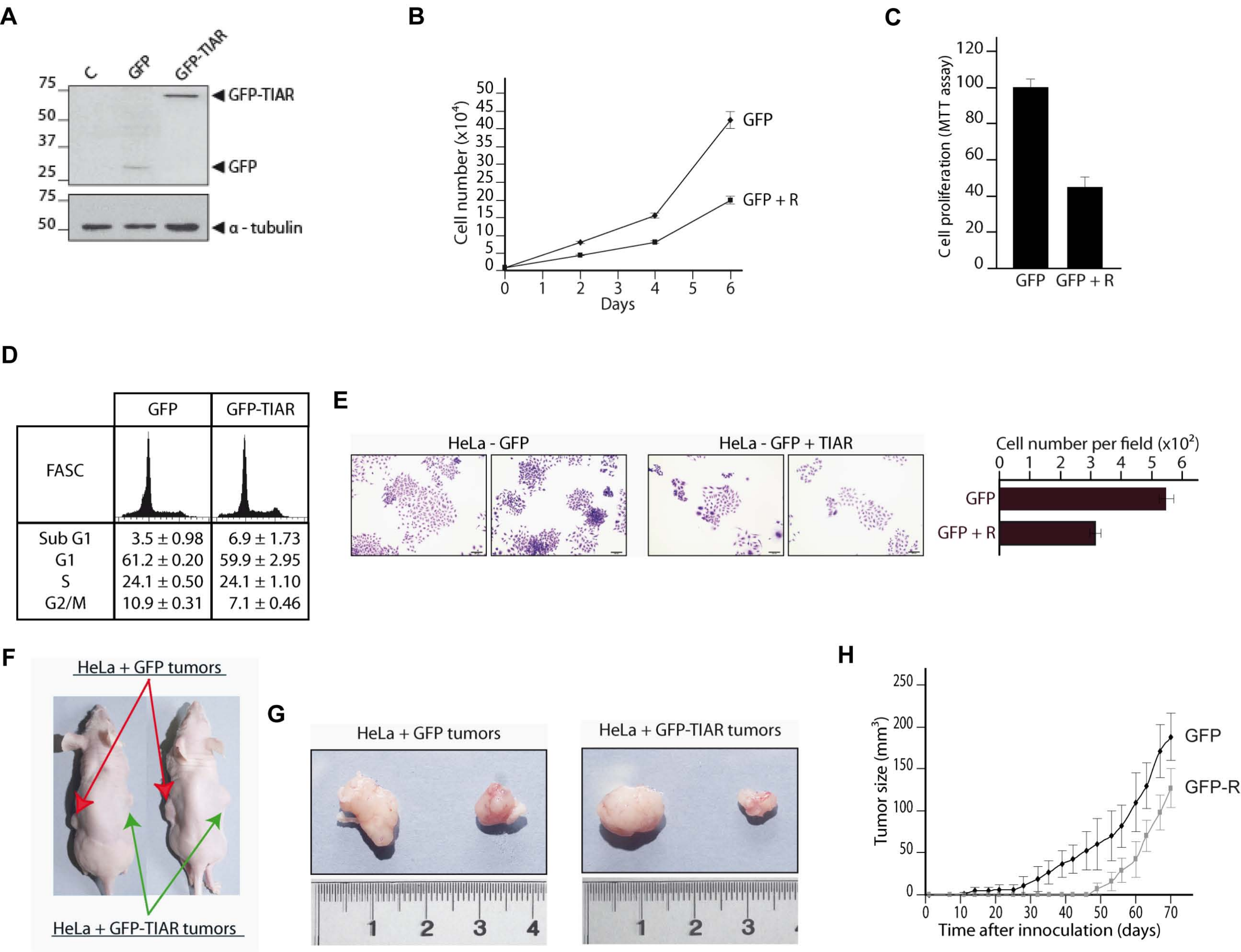

Supplement: Supplementary Information [file cddis201543x1.pdf]
